# Oxidative changes and signalling pathways are pivotal in initiating age-related changes in articular cartilage

**DOI:** 10.1136/annrheumdis-2014-206295

**Published:** 2015-01-28

**Authors:** Wang Hui, David A Young, Andrew D Rowan, Xin Xu, Tim E Cawston, Carole J Proctor

**Affiliations:** 1 MRC/Arthritis Research UK Centre for Musculoskeletal Ageing (CIMA), Musculoskeletal Research Group, Institute of Cellular Medicine, Medical School, Newcastle University, Newcastle upon Tyne, UK; 2 Biomedicine Biobank, Institute of Cellular Medicine, Medical School, Newcastle University, Newcastle upon Tyne, UK; 3 Newcastle University Institute for Ageing, Newcastle University, Campus for Ageing and Vitality, Newcastle upon Tyne, UK

**Keywords:** Chondrocytes, Osteoarthritis, Synovial fluid

## Abstract

**Objective:**

To use a computational approach to investigate the cellular and extracellular matrix changes that occur with age in the knee joints of mice.

**Methods:**

Knee joints from an inbred C57/BL1/6 (ICRFa) mouse colony were harvested at 3–30 months of age. Sections were stained with H&E, Safranin-O, Picro-sirius red and antibodies to matrix metalloproteinase-13 (MMP-13), nitrotyrosine, LC-3B, Bcl-2, and cleaved type II collagen used for immunohistochemistry. Based on this and other data from the literature, a computer simulation model was built using the Systems Biology Markup Language using an iterative approach of data analysis and modelling. Individual parameters were subsequently altered to assess their effect on the model.

**Results:**

A progressive loss of cartilage matrix occurred with age. Nitrotyrosine, MMP-13 and activin receptor-like kinase-1 (ALK1) staining in cartilage increased with age with a concomitant decrease in LC-3B and Bcl-2. Stochastic simulations from the computational model showed a good agreement with these data, once transforming growth factor-β signalling via ALK1/ALK5 receptors was included. Oxidative stress and the interleukin 1 pathway were identified as key factors in driving the cartilage breakdown associated with ageing.

**Conclusions:**

A progressive loss of cartilage matrix and cellularity occurs with age. This is accompanied with increased levels of oxidative stress, apoptosis and MMP-13 and a decrease in chondrocyte autophagy. These changes explain the marked predisposition of joints to develop osteoarthritis with age. Computational modelling provides useful insights into the underlying mechanisms involved in age-related changes in musculoskeletal tissues.

## Introduction

Cartilage is a unique tissue in which the sole cell type, the chondrocyte, precisely arranges extracellular matrix (ECM) macromolecules, which mainly consists of type II collagen and aggrecan, to underpin normal tissue function and architecture. In normal cartilage, chondrocytes maintain a dynamic equilibrium with a balance between ECM production and its proteolytic breakdown. Chondrocytes secrete cartilage-degrading enzymes such as collagenases, members of the matrix metalloproteinase (MMP) family which are the most effective proteolytic enzymes at cleaving native collagen during the loss of cartilage that characterises osteoarthritis (OA).^1^ OA is characterised by a disruption of the articular cartilage surface^2^ and MMP-13 is a key mediator within OA cartilage that plays a significant role in cartilage collagen breakdown.^3^ ^4^

Multiple genetic and environmental factors are implicated in the development of OA but ageing is the most important risk factor.^5^ A variety of changes occur in cartilage with age that include an accumulation of oxidative stress, DNA and protein damage, overproduction of proteolytic enzymes by chondrocytes, a loss of cartilage matrix and a decrease in the ability of chondrocytes to function normally and to survive.^6^ ^7^ Autophagy is protective in normal cartilage and loss of this mechanism with age increases cell death associated with OA.^8^ ^9^ Apoptosis also contributes to the loss of cellularity and cartilage degeneration in OA with a central role for the caspase proteolytic cascade.^10^ However, the relationship between these mediators, how they contribute to age-related changes in cartilage and the cartilage degeneration seen in disease remains to be determined.

The aim of this study was to rigorously assess the changes and sequence of events that occur within joints taken from mice aged from 3 to 30 months with respect to morphology, cellular changes, matrix loss and the presence of relevant mediators with a view to identifying mechanisms that predispose aged cartilage to degeneration and the development of OA. Combined with this, we have used computational modelling to aid our understanding of the age-related processes^11^ by integrating the different mechanisms of ageing identified in the histological study to provide a robust and testable model of the underlying mechanisms and the interplay between them.

## Methods

### Animals

Mice were from a long-established colony of the inbred C57/BL1/6 (ICRFa) mouse strain selectively bred for longevity.^12^ Both knee joints were collected from male mice aged 3–30 months (four mice per group). Mice were housed in standard cages in groups of four to six which did not change from weaning. Mice were provided with *ad libitum* food and water and housed at 20±2°C under a 12 h light/12 h dark photoperiod. Procedures were performed in accordance with the UK Home Office regulations.

### Reagents

Polyclonal antibodies to MMP-13 were raised in rabbit.^13^ Anti-type-II collagen collagenase cleavage site neoepitope antibody (COL2-1/4N1) was a gift from E. Lee (Shriner's Hospital for Children, Montreal, Canada).^14^ Anti-3-nitrotyrosine antibody (ab61392) was from Abcam, Cambridge, UK; anti-LC-3B antibody (L7543) was from Sigma-Aldrich, Poole UK; anti-Bcl-2 (PC68) and anti-Bax (PC66) rabbit polyclonal antibody were purchased from Calbiochem, Germany; activin receptor-like kinase-1 (ALK1) (C-20:SC-19547) and transforming growth factor-β (TGF-δ) RI(V-22;sc-398) were purchased from SANTA CRUZ Biotechnology. VECTASTAIN Elite ABC kits PK 6102 and 6106 were from Vector Laboratories (Burlingame, California, USA). All other reagents were commercially available analytical grade obtained from Sigma-Aldrich.^13^

### Histological assessment of OA changes with age in mice knee joints

Histology was performed as described.^13^ Knee joints were fixed in 4% paraformaldehyde solution for 24 h then decalcified in 10% (w/v) EDTA in phosphate buffer for 10 days. Joints were embedded in paraffin and frontal sections (5 µm) cut across each entire joint, followed by staining with Weigert's haematoxylin and Safranin-O/Fast Green. Multiple sections of each entire joint were graded using Osteoarthritis Research Society International scoring system, by two closely correlating scorers blinded to the specimens.^15^ The higher scores for each joint were used to populate data samples for each joint.

### Immunohistochemistry

Sections were analysed using immunohistochemistry as described.^13^ Formalin-fixed paraffin sections were deparaffinised, rehydrated and treated with 0.05% (w/v) trypsin (ll-S, Sigma) at 37°C for 20–30 min. Sections were blocked (1.5% normal sheep serum) for 30 min, and then incubated with primary antibodies at the dilutions stated: anti-COL2-1/4N1 (1:1500), anti-MMP-13 (1:250), anti-nitrotyrosine (1:2000), Bcl-2 (1:40), anti-ALK1 (1:100), anti-ALK5 (1:200), anti-LC-3B (1:150 ) and normal rabbit immunoglobulin G (as an isotype-matched control) overnight at 4°C. After sequential incubations with biotinylated secondary antibody and avidin–biotin complex using the Vectastain kit 6101 (Vector, Peterborough), signal was developed using 3,3′-diaminobenzidine tetra-hydrochloride chromogenic solution (DAKO, Ely, UK) with haematoxylin counterstaining.^13^

### Quantification of chondrocyte immunostaining in sections from joints of aged mice

Positively stained chondrocytes in knee articular cartilage from C57/BL (ICRFa) mice were counted by two blinded observers. The number of immunopositive cells were counted in each section and expressed as a percentage of the total number of cells with a minimum of 100 cells counted each time.

### Image and statistical analyses

Images of stained sections were captured using a Leica DMR microscopy with the Leica DFC310 FX 1.4-megapixel digital colour camera (Leica Microsystems, Wetzlar, Germany). Student t test was used for statistical analysis. p Values <0.05 were considered significant.

### Model construction

**Figure 1:**
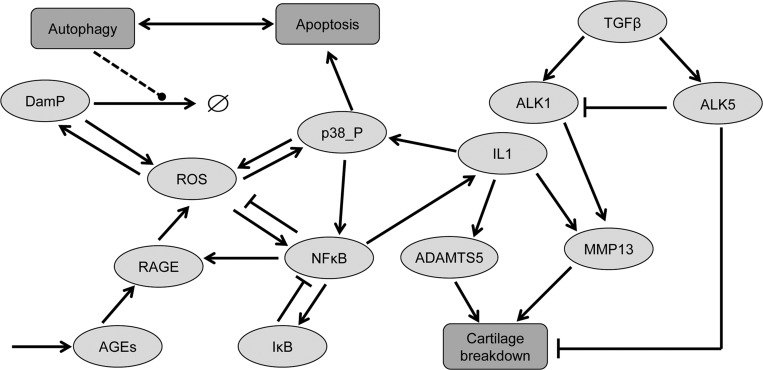
Simplified diagram of the model network showing the main components in the model and how they interact. Advanced glycation end products (AGEs) are produced spontaneously and activate RAGE receptors. Reactive oxygen species (ROS) are produced by activated RAGE receptors and damaged protein (DamP) in addition to basal ROS generation via mitochondria (not shown). DamP may be degraded by lysosomes. ROS increases damage to protein, increases phosphorylation of p38MAPK and activates nuclear factor (NF)-κB. NF-κB upregulates superoxide dismutase to inhibit ROS, upregulates IκB to inhibit itself and activates interleukin (IL) 1. IL-1 upregulates ADAMTS-5 and matrix metalloproteinase-13 (MMP-13) which degrades aggrecan and collagen II, respectively. MAPK p38 leads to activation of apoptosis. There is also crosstalk between autophagy and apoptosis via Beclin, Bcl2, Bax and caspases (not shown). Transforming growth factor-β (TGF-β) normally signals via activin receptor-like kinase-5 (ALK5) to upregulate genes involved in cartilage synthesis. ALK1 signalling increases with age and leads to upregulation of MMP-13 and so exacerbates cartilage breakdown. Detailed figures showing all the components in the signalling pathways are shown in online supplementary figures S1–S5.

A computational model was constructed to incorporate the age-related changes observed in the mouse joints. We included components that had been directly measured and others known to be important in ageing. These were chosen as a result of a literature search on ageing and OA which indicated that key components included the molecular mechanisms involved in oxidative stress,^16^ protein damage,^17^ accumulation of advanced glycation end-products,^18^ autophagy,^9^ apoptosis,^19^ nuclear factor (NF)-κB signalling,^20^ TGF-β signalling,^21^ upregulation and activation of matrix-degrading enzymes^22^ and cartilage turnover (figure 1). Because the model is complex, we split it into five modules: damage, NF-κB, TGF-β/Alk1, TGF-β/Alk5 and autophagy/apoptosis. The model assumptions are given in the online supplementary file together with full details of all components and reactions (see online supplementary figures S1–S5 and tables S1–S6). The model was constructed as a biochemical network using CellDesigner^23^ and encoded in the Systems Biology Markup Language.^24^ Stochastic simulations were performed in COmplex PAthway SImulator^25^ and on a computer cluster using code developed by Newcastle University.^26^ Results were analysed in R and plotted with the R package ggplot2.^27^ The model was deposited in BioModels Database^28^ and assigned the identifier (MODEL1402200004).

## Results

### Age-related changes in knee joint morphology

**Figure 2:**
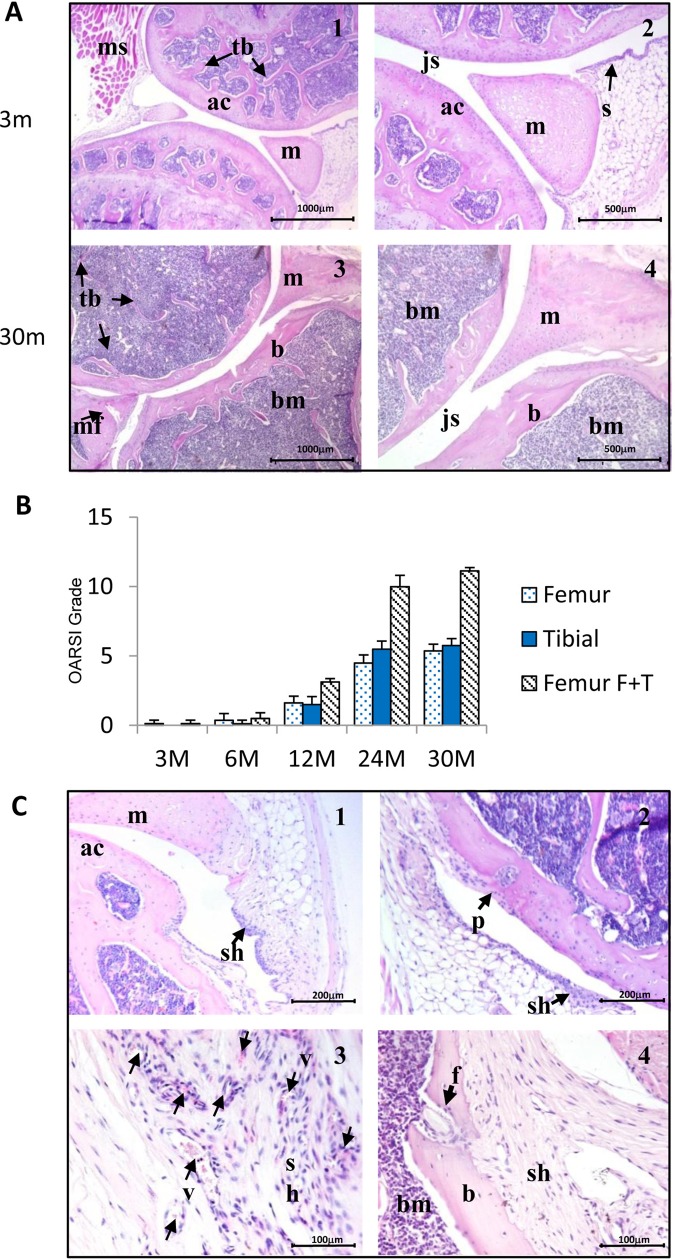
Age-related changes in the morphology of mouse knee joints. Knee joints were collected and stained with H&E. At 3 months (A1, A2), the joint surfaces were covered by normal articular cartilage, but by 30 months, a profound loss of cartilage was seen (A3, A4). Semiquantitative scoring of the joints indicated the development of severe osteoarthritis in aged mice (B). No noticeable defects were observed at 3 and 6 months but sections were scored grade 1–2 at 12 months and 5–6 at 24 and 30 months. Data are expressed as the mean±SD. Statistical significance: **p<0.01 versus the 3-month-old group. In some knee joints significant lesions were observed. At 12 months there was evidence of increased synovial cell hyperplasia (C1) and evidence of invasion of synovial cells into the articular cartilage (C2); top arrow indicates site of erosion. At 30 months (C3), blood vessels were observed (small arrows) within the synovium and bone fracture caused by synovial cell invasion (C4) was seen in some joints. ac, articular cartilage; b, bone; bm, bone marrow; f, bone fracture; js, joint space; m, meniscus; mf, meniscus fracture; ms, muscle; OARSI, Osteoarthritis Research Society International; p, pannus; s, synovium; sh, synovial hyperplasia; tb, trabecular bone; v, blood vessel. All mice were male, n=4.

Knee joints from 3-month-old mice showed a smooth and organised cartilage surface, clear joint space and normal synovium (figure 2A1, A2). There was progressive loss of joint space and synovial changes with age. At 30 months, joints showed cartilage surface discontinuity, vertical fissures to the cartilage/bone interface, substantial loss of articular cartilage in the midline of the joint and thinning of the subchondral bone (figure 2A3, A4). Semiquantitative scoring indicated the development of severe OA in aged mice: joint sections were scored grades 1–2 at 12 months and 5–6 at 24 and 30 months (figure 2B).

There was loss of cellularity in the aged cartilage samples compared with young mice, and in some joints a significant increase was observed in synovial hyperplasia by 12 months (figure 2C1) and there was evidence of localised invasion into cartilage (figure 2C2). At 30 months, an increase in synovial blood vessel formation was often observed (figure 2C3), and sometimes bone fracture was seen where synovial cells appeared to invade (figure 2C4).

### Age-related loss of cartilage matrix proteins

**Figure 3:**
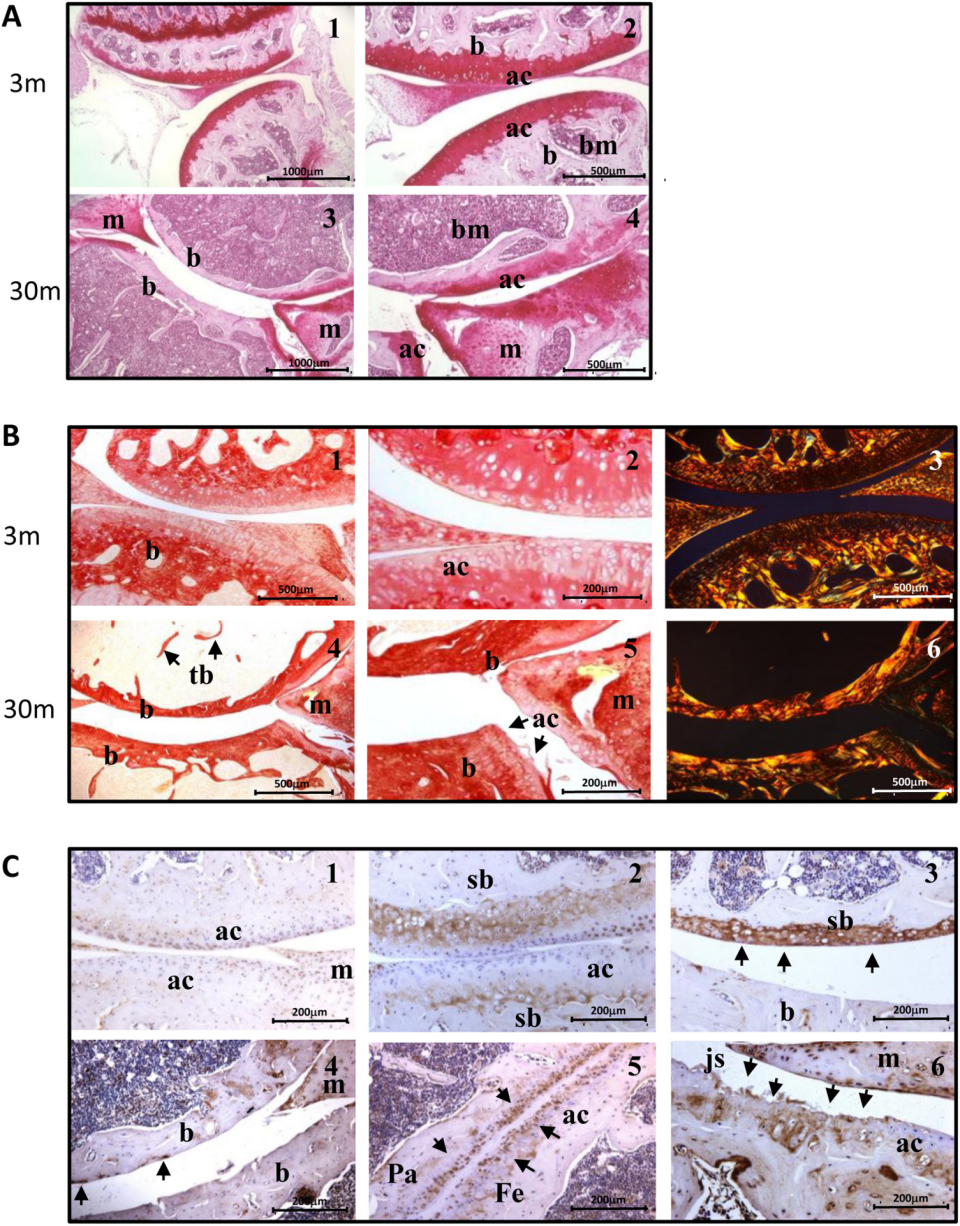
Age-related changes in proteoglycan, collagen and matrix metalloproteinase (MMP)-specific collagen cleavage in mouse knee joint cartilage. Knee joints were sectioned and stained for proteoglycan by Safranin-O, collagen with Sirius Red and immunostained for the MMP-cleavage site of type II collagen. Strong proteoglycan staining was seen at 3 months (A1,A2) but there was progressive proteoglycan loss with complete removal of cartilage at the midline of the joint at 30 months (A3,A4). Strong collagen staining was seen in cartilage at 3 months (B1,B2). Under polarised light (B3) collagen fibres appeared bright yellow or orange with reticular fibres staining green. Collagen was reduced with age in the superficial and middle zones, and by 30 months, the cartilage was completely removed from the midline of the joint (B4,B5,B6). Little specific collagen cleavage was seen at 3 months (C1), this was increased at 12 months in the middle and deep layers of cartilage (C2). At 24 months, increased staining was seen (C3), and by 30 months, almost complete loss of articular cartilage was observed with only small isolated pockets stained (C4). In some joints increased CII staining was seen at cartilage surfaces (C5) with localised staining at cartilage erosion sites at the margins of the joint at 30 months (C6). ac, articular cartilage; b, bone; bm, bone marrow; Fe, femur; js, joint space; m, meniscus; Pa, patella; sb, subchondral bone; tb, trabecular bone. All mice were male, n=4.

The proteoglycan content of young (3 months) joints (figure 3A1, A2) showed strong staining. However, with increasing age, there was progressive loss of proteoglycan initially at the surface. At 30 months, substantial loss of proteoglycan staining was seen in all zones of cartilage, with staining seen only at the joint margins and within the meniscus (figure 3A3, A4).

There was progressive loss of collagen with age in articular cartilage (figure 3B). At 3 months, well-organised lacunae containing chondrocytes were seen in the superficial and middle zones (figure 3B1, B2). Visualisation by polarised light indicated a clearly delineated smooth articular surface at 3 months with the orange-stained collagen fibres orientated in a radial direction to the cartilage surface with small dark areas indicating high cellularity within the cartilage at the superficial and mid-zone (figure 3B3). By 30 months (figure 3B4, B5) there was significant loss of surface cartilage with complete exposure of the subchondral bone at the midline of the joint plus changes to the subchondral bone. Any cartilage remaining was confined to the margins of the joint. Visualisation using polarised light revealed that at 30 months (figure 3B6) there was substantial loss of collagen with thinning of the remaining, less organised collagen fibres.

### Age-related increase in type II collagen cleavage

An increase in the specific MMP-cleavage of collagen type II was observed with age. Low levels were detected in young cartilage (figure 3C1) but this increased with age and by 12 months increased levels of CII cleavage were observed (figure 3C2) especially in the middle or deep layers of cartilage and at the femoral–patellar joint margins. At 24 months, increased staining was observed with significant loss of articular cartilage especially on the tibial surface (figure 3C3). Strong staining was found at isolated sites of erosion at 30 months (figure 3C4, C5) with most of the cartilage lost from the articular surface and staining confined to isolated sites of any remaining cartilage (figure 3C6).

### Age-related increase in the expression of MMP-13

**Figure 4:**
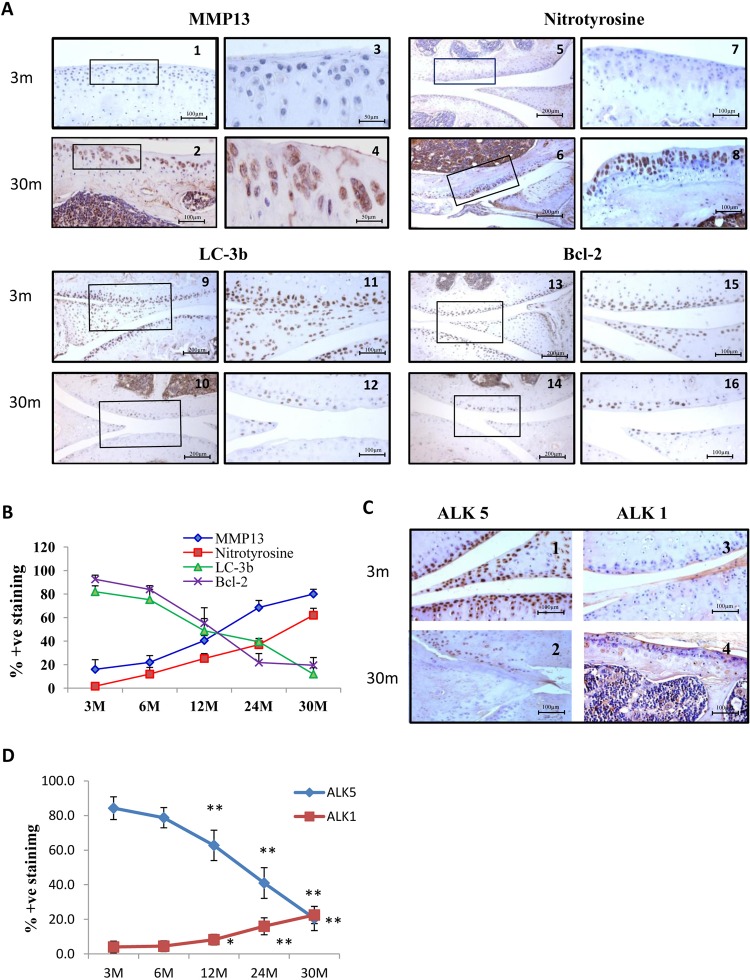
Age-related changes in matrix metalloproteinase (MMP)-13, nitrotyrosine, LC-3B, Bcl-2, activin receptor-like kinase-5 (ALK5) and ALK1 expression in mouse knee joint articular cartilage. Knee joints were sectioned and immunostained with antibody to MMP-13, nitrotyrosine, LC-3B, Bcl-2, ALK5 or ALK1 and counterstained with haematoxylin. At 3 months, little staining was seen for MMP-13 but substantial staining was observed at 30 months (A1–A4). MMP-13 was significantly raised at all time points from 12 months (B). Little staining for nitrotyrosine was seen at 3 months with increased staining at 30 months (A5–A8). Nitrotyrosine was significantly raised at each age group (B). Strong staining was observed for LC-3B at 3 months with little staining at 30 months (A9–A12) with LC-3B significantly increased at 3–24 months compared with 30 months (B). Strong staining of Bcl-2 was seen at 3 months with less staining observed at 30 months (A13–A16) with Bcl-2 significantly higher at 3–12 months compared with 30 months (B). High levels of ALK5 were present at 3 months but this was not found at 30 months (C1, C2) with ALK5 significantly lower at 12–30 months compared with 3 months (D). ALK1 was present at low levels at 3 months with higher levels at 30 months in cartilage and within synovial tissues (C3, C4) with ALK1 significantly higher at 12–30 months compared with 3 months (D). Graphical data (B) are expressed as the mean±SD. Statistical significance: MMP-13: 12, 24, 30 months versus 3 m, p value<0.01; nitrotyrosine: 6 months versus 3 months, p value <0.05; 12, 24, 30 months versus 3 months, p value <0.01; LC-3B: 3, 6, 12 and 24 months versus 30 months, p value <0.01; Bcl-2: 3, 6, 12 months versus 30 months, p value <0.01. All mice were male, n=4.

MMP-13 is often detected at sites of joint damage and is strongly implicated in cartilage destruction in OA. We investigated if the increase in CII cleavage was accompanied by a corresponding increase in MMP-13. There was little MMP-13 staining in cartilage in mice at 3 months; the proportion of MMP-13-positive chondrocytes increased with time and at 30 months significant staining of chondrocytes was seen (figure 4A1–A4). Less than 16.00±8.29% of chondrocytes were positive for MMP-13 at 3 months and this rose to 80.00±4.08% of cells at 30 months. The numbers of MMP-13 positive chondrocytes were significantly increased (p<0.01) at 12, 24 and 30 months compared with 3-month cartilage (figure 4B). These data support a role for MMP-13 in the observed cleavage of type II collagen in ageing cartilage (figure 3C).

### Age-related increase in the expression of nitrotyrosine

There was little nitrotyrosine staining in cartilage of 3-month-old mice which progressively increased by 30 months (figure 4A5–A8). Nitrotyrosine levels were significantly raised (p=0.0122) as early as 6 months rising to 62±5.88% at 30 months (figure 4B).

### Age-related decrease in the expression of LC-3B

Autophagic dysfunction is associated with ageing and human disease, including OA.^29^ Figure 4A9–A12 illustrates that high levels of staining for LC-3B were observed at 3 months but this was much reduced at 30 months. At 3 months, 82±5.0% of chondrocytes in the superficial and mid layers of cartilage expressed LC-3B. This declined with age and by 30 months only 12.00±5.4% of chondrocytes expressed LC-3B (figure 4B), suggesting that autophagic dysfunction is indeed associated with age in cartilage.

### Age-related decrease in the expression of Bcl-2

Overexpression of Bcl-2 is known to suppress apoptosis and high levels of Bcl-2 were found in mouse joint cartilage at 3 months. This was significantly decreased by 30 months (figure 4A13–A16) suggesting that apoptosis of chondrocytes also increases with age in murine cartilage. Figure 4B illustrates that at 3 months 60.50±8.2% of chondrocytes expressed Bcl-2 but this was progressively reduced with age with 32.50±5.3% and 18.25±3.3% of chondrocytes expressing Bcl-2 at 24 and 30 months, respectively (p<0.01; 30 vs 3 months).

### Age-related changes in the levels of the TGF-β receptors ALK1 and ALK5

Because alterations in the levels of ALK1 and ALK5 have been reported in human OA,^21^ we investigated the levels of these TGF-β receptors in aged murine cartilage. High levels of ALK5 were seen at 3 months, which were much reduced by 30 months (figure 4C1, C2, D). Conversely, ALK1 levels were relatively low at 3 months but increased with age (figure 4D, C3, C4).

### Computational modelling of the age-related changes in cartilage

**Figure 5:**
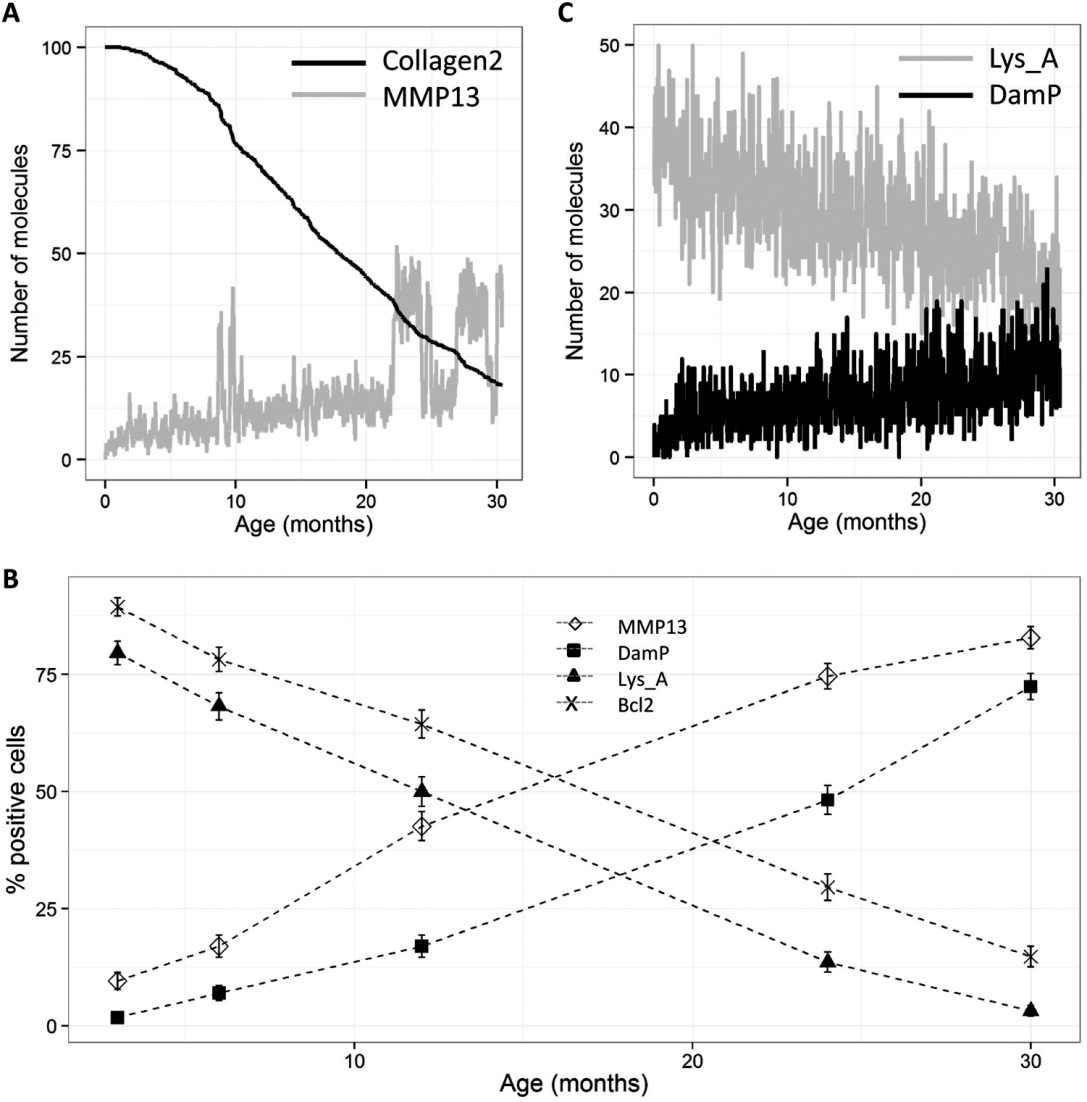
Simulation results showing changes in collagen II, matrix metalloproteinase-13 (MMP-13) active lysosomes (Lys_A) and damaged protein (DamP) with age. (A) One randomly chosen simulation run (from 500 stochastic simulations) shows percentage of collagen II and levels of MMP-13. (B) The percentage of ‘simulated cells’ expressing MMP-13 (>15), DamP (>9), Lys_A (>29) and Bcl2 (>12). (C) One randomly chosen simulation run (from 500 stochastic simulations) shows levels of DamP and Lys_A. Error bars in B represent a 95% CI of the percentage.

The computational model of ageing cartilage (figure 1) was parameterised to fit the histochemical data. The model output showed a progressive loss in cartilage collagen (figure 5A) with a mean percentage loss of 80.5%. The model includes upregulation of MMP-13 by both the interleukin 1 (IL-1) and TGF-β/ALK1 pathways and the model output showed an increase in MMP-13 levels with age (figure 5A). The small, gradual increase over time was due to IL-1 activation and the large intermittent peaks of MMP-13 were a result of Runx2 activation via ALK1 signalling. In most simulations, these peaks occurred more frequently at later time points due to the increase in the ALK1/ALK5 ratio with age. We calculated the percentage of ‘simulated cells’ from 500 stochastic simulations that expressed MMP-13 above a basal level of 15 molecules (figure 5B). The model output closely matched the experimental data (figure 3B).

A gradual decline in lysosome activity (modelled by assuming that lysosomes require Beclin for activation) and an increase in damaged protein over time was observed in individual simulations (figure 5C) and the percentage of ‘simulated cells’ expressing activated lysosomes decreased, and damaged proteins increased with age (figure 5B), similar to the experimental data (figure 3B). The model output showed a decline in Bcl-2 levels with age (data not shown) and the percentage of ‘simulated cells’ expressing Bcl-2 above a threshold level declined with age (figure 5B). The model also showed an increase in caspase activation at later time points (data not shown) suggesting that levels of apoptosis increase with age.

### Computational modelling supports an important role for oxidative stress and the IL-1 pathway

**Figure 6:**
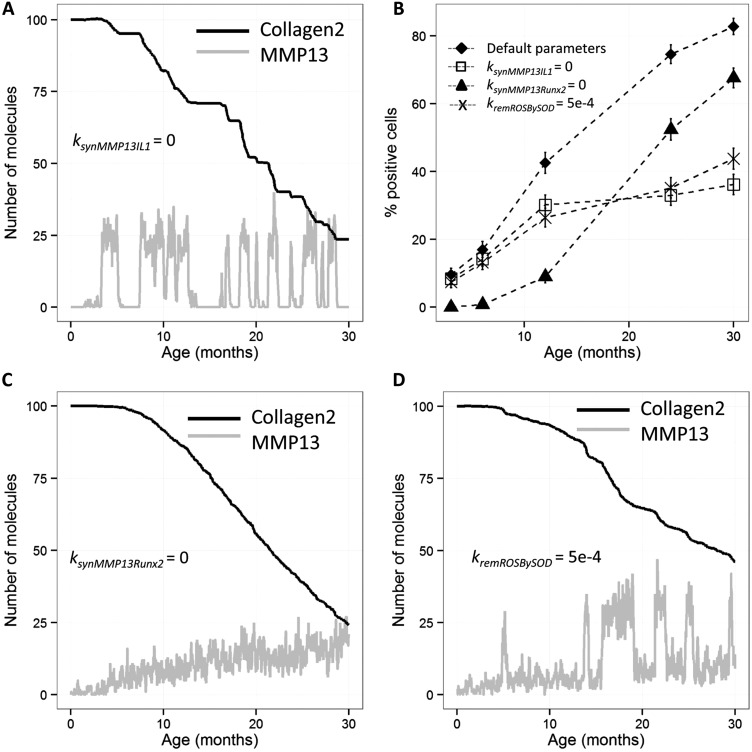
Computer simulation results obtained from varying three of the model parameters. In each computer experiment, 500 stochastic simulations were run. (A) Total inhibition of matrix metalloproteinase-13 (MMP-13) synthesis via interleukin-1 pathway (ksynMMP13=0). (B) The percentage of cells expressing MMP-13 (>15) for the different parameter sets. (C) Total inhibition of MMP-13 synthesis via ALK1 pathway (ksynMMP13Runx2=0). (D) Increased removal of reactive oxygen species by superoxide dismutase (kremROSBySOD=5e-4/molecules/s). A, B, and D Output from one randomly selected simulation, black curves show level of collagen II molecules (as a percentage), grey curves show level of MMP-13. Error bars in B represent a 95% CI of the percentage.

By varying some of the model parameters, we were able to gain some insights into which pathways particularly lead to upregulation of MMP-13 and cartilage degradation. To examine the relative contribution of the IL-1 and TGF-β/ALK1 pathways, we inhibited MMP-13 upregulation by each pathway in turn. Total inhibition of MMP-13 synthesis via the IL-1 pathway led to lower levels of MMP-13 at later time points and a delay in cartilage degradation (figure 6A, B). The intermittent peaks of MMP-13, due to the TGF-β/ALK1 pathway, accounted for the early degradation of cartilage. Conversely, total inhibition of MMP-13 synthesis via the TGF-β/ALK1 pathway led to lower levels of MMP-13 at early time points but did not prevent the age-related increase in MMP-13 and the rate of cartilage degradation was not reduced (figure 6B–C). Because oxidative stress was an important mechanism included in the model, we examined the effect of increased reactive oxygen species removal via superoxide dismutase (SOD). This simulated intervention led to lower levels of MMP-13 via the IL-1 pathway, especially at late ages, and cartilage degradation was reduced (figure 6B, D).

## Discussion

Ageing is the most significant risk factor for the development of OA and until recently little has been known of the age-related changes that occur in cartilage. In this study, we have investigated the cellular and matrix changes that occur with age in the knee joints of male mice from an established and well-characterised aged mouse colony from 3 to 30 months.^30^ Some of the observed changes have been documented before, but to date, this is the first study to carry out multiple measures in a set of tissues from an aged mouse colony to enable a better understanding of the interplay between underlying complex mechanisms. We demonstrated an overall loss of cartilage matrix with evidence of type II collagen degradation alongside increased levels of MMP-13, the major collagenase in cartilage turnover, and evidence of increased levels of products of oxygen radicals with age. Cellular changes included a decrease in chondrocyte autophagy and a decrease in a marker indicative of less suppression of cell death via apoptosis. Moreover, evidence that chondrocyte cell death correlates with progression of OA in mice^31^ further supports out findings. Interestingly, it has also been shown that an increase in apoptosis in OA is related to a decline in autophagy in concordance with our analysis.^9^ Moreover, chondrocyte hypertrophy in OA has been previously reported, occurring at different times during disease progression and at different locations.^32^ Although we did not assess hypertrophy, such cellular changes will undoubtedly contribute to the age-related cartilage changes we observed. Another limitation of the study is that the MMP-13 antibody stained both pro-MMP-13 and active MMP-13, but we also showed that collagen cleavage occurred, indicating that MMP-13 must be active.

It has been proposed that young chondrocytes are held in a quiescent state by TGF-β acting through the receptor ALK5.^33^ With age the levels of this receptor decrease, the ratio of ALK1 to ALK5 increases, so that TGF-β signals predominantly through ALK1. As a result, the chondrocytes develop an autolytic phenotype that degrades the surrounding matrix. In young cartilage TGF-β is protective but with increased signalling through ALK1, MMP-13 is upregulated and cartilage breakdown ensues. These data fit with the increased expression of ALK1, MMP-13 and CII cleavage with age found in the current study and so were incorporated in the computational model.

Loeser^34^ described a senescent secretory phenotype of chondrocytes present in OA induced by random genomic damage following increased oxidative stress and a decline in proliferative and synthetic capacity with age. We observed an increase in nitrotyrosine levels with age, the levels of 8-oxo DNA are known to increase in OA cartilage, and we have previously described a decrease in SOD to precede damage in OA cartilage with resultant dysfunctional mitochondria.^35^ An increase in proinflammatory mediators and matrix-degrading enzymes with an increasingly resistant matrix that all contribute to the development of disease have been described;^34^ we also observed such changes.

The integrative computational model of the molecular mechanisms of ageing was able to reproduce the age-related changes in cartilage and provide a novel way of identifying interactions between different components. We incorporated mechanisms that were highlighted in this study or have previously shown to be important in cartilage degradation but it has been constructed in a format that can be readily extended by ourselves and others as required. Thus, the model is able to integrate information from different sources and provide a useful tool for gaining insights into the key mechanisms driving the age-related changes in cartilage.

Several transcriptomic studies have been performed examining changes in gene expression in murine OA pathogenesis.36–38 Loeser *et al*^37^ showed age-related differences in gene expression in both sham-operated control and surgically induced OA mice and demonstrated the importance of ageing in OA animal model studies confirming the need to study age-related changes in long-lived mice. Interestingly, another transcriptomic study^38^ showed that chondrocytes in articular cartilage of OA mice had similar gene expression profiles with skeletal muscle which was associated with inappropriate NF-κB signalling, supporting findings from our computational model. The advantage of transcriptomic approaches is that new genes and pathways can be identified and thus it is possible to examine how gene expression is affected by nodes in the network. For example, Bateman *et al*^36^ compared profiles from wild-type mice with mice lacking ADAMTS-5 activity and identified several genes that have an ADAMTS-5 independent role in OA. Proteomic studies are also important as changes in protein expression with age do not always correlate with gene expression although this method is still fairly new in OA research.^39^ The disadvantage of ‘omic’ approaches is that it can be difficult to work out the actual detailed mechanisms involved in initiation and progression of OA. This highlights the merit of computational models which are able to examine detailed mechanisms, simulate the temporal effects of single or multiple interventions and make testable predictions. Both approaches should be considered complementary as new information on genes and pathways gathered from transcriptomic and proteomic approaches can be used to modify and extend the current model.

We have demonstrated that a progressive loss of cartilage matrix and cellular changes occur with increasing age accompanied by an increase in the level of oxidative stress and MMP-13 in murine articular cartilage. We modelled these changes using a systems biology approach and demonstrated that oxidative damage and the IL-1 pathway are pivotal in initiating the changes that lead to the development of disease. Interestingly, the model predicts that blocking the IL-1 pathway early in disease is effective at blocking MMP-13 production while blocking ALK1-mediated MMP-13 production is much more effective in aged tissues. This finding may have implications for therapeutic interventions. It would be interesting to test the model predictions by the use of inhibitors, and the use of an OA-prone mouse model such as STR/Ort would be ideal for such future studies.

## Supplementary Material

Web supplement
