# Supplementary material for: Oxidative changes and signalling pathways are pivotal in initiating age-related changes in articular cartilage
Source: Ann Rheum Dis. 2015 Jan 28;75(2):449–58. doi: 10.1136/annrheumdis-2014-206295 (PMC4752670; doi:10.1136/annrheumdis-2014-206295)
Supplement: Web supplement [file annrheumdis-2014-206295-s1.pdf]

Oxidative changes and the IL-1 pathway are pivotal in initiating age-related changes in articular cartilage

Wang Hui[a], David A. Young[a], Andrew D. Rowan[a], Xin Xu[b], Tim E. Cawston[a] and Carole J. Proctor[a,c]

[a]MRC/Arthritis Research UK Centre for Musculoskeletal Ageing (CIMA), Musculoskeletal Research Group, Institute of Cellular Medicine, Medical School, Newcastle University, Newcastle upon Tyne NE2 4HH, United Kingdom. [b]Biomedicine Biobank, Institute of Cellular Medicine, Medical School, Newcastle University, Newcastle upon Tyne NE2 4HH, United Kingdom. [c]Institute for Ageing and Health, Newcastle University, Newcastle upon Tyne, NE4 5PL, United Kingdom.

Supplementary Text

Model assumptions 2

Supplementary Figures

Figure S1 Damage module 5

Figure S2 NFκB module 6

Figure S3 TGFβ/Alk5 module 7

Figure S4 TGFβ/Alk1 module 8

Figure S5 Autophagy/Apoptosis module 9

Supplementary Tables

Table S1 List of model species 10

Table S2 Reactions for Damage module 11

Table S3 Reactions for NFκB module 12

Table S4 Reactions for TGFβ/Alk5 module 12

Table S5 Reactions for TGFβ/Alk1 module 13

Table S6 Reactions for Autophagy/Apoptosis module.. 14

References 14

Model Assumptions

Damage module

The components and reactions for this module are shown in Figure S1. Advanced glycation end-products are spontaneously produced at a very low rate but accumulate with age. They activate RAGE receptors which increase levels of reactive oxygen species (ROS) [1]. It is assumed that there is a constant pool of native protein (NatP). ROS cause damage to proteins, lysosomes (leading to inhibition of their activity), and activates p38.[2] Damaged protein is removed by activated lysosomes. RAGE receptors also activate NFκ B[1] (see Figure S2) which increases levels of the antioxidant superoxide dismutase (SOD) and so helps to reduce ROS. Aggrecan protects collagen 2 from degradation.[3] We model this by assuming that aggrecan forms a complex with collagen 2 to protect it from the activity of collagenases. . ADAMTS-5 cleaves aggrecan to produce aggrecan fragments,[4] which then exposes collagen 2 so that it can by cleaved by the collagenase MMP-13 to produce collagen fragments.[5]

NFκB module

The components and reactions for this module are shown in Figure S2. NFκB is normally inactive by being in complex with IκB. Under stress conditions IκB is degraded releasing NFκB; in the model this can be catalysed by IL-1 or ROS.[6] IL-1 signalling leads to upregulation of ADAMTS-5, MMP-2 and MMP-13, and phosphorylation of p38.[7] Phospho-p38 phosphorylates NFκB to activate its transcriptional activity.[8] We include a small subset of target genes including IL-1, IκB, RAGE and SOD. For simplicity, the model does not include detail of transcription and translation so that protein synthesis is shown by a single reaction.

TGFβ/Alk5 module

The components and reactions for this module are shown in Figure S3. TGFβ is normally in the extra-cellular matrix in an inactive state. It can be activated by a mechanical stimuli

(represented by Integrin in the model) or MMP-2.[9] To model infrequent and transient mechanical stimuli we assume that Integrin synthesis occurs at a very low rate and is then degraded very quickly. During the short time period when Integrin is present it can activate TGFβ. TGFβ signals via the Alk5 pathway by binding to Alk5 dimers. This leads to phosphorylation of Smad2 which then binds Smad4 to form a complex. This complex activates Sox9 and also directly upregulates collagen 2 and Smad7. Activated Sox9 upregulates aggrecan, collagen2 and Sox9. The Smad2/Smad4 complex also activates upregulation of Smad7. Smad7 binds to the TGFβ/Alk5 complex which leads to degradation of Alk5 and Smad7 itself.[10]

TGFβ/Alk1 module

The components and reactions for this module are shown in Figure S4. TGFβ also signals via the Alk1 pathway but this also requires Alk5.[11] This is modelled by assuming that Alk1 forms a heterodimer with Alk5 before TGFβ can bind. The TGFβ/Alk1/Alk5 complex activates Smad1 by phosphorylation. Phospho-Smad1 binds to Smad4 and upregulates Runx2 which then leads to upregulation of MMP-13. We also assume that the Smad2/Smad4 complex inhibits Runx2 activity.[12] Smad7 may also bind to the TGFβ/Alk1/Alk5 complex leading to degradation of Alk1, Alk5 and Smad7 itself. Smad7 also prevents activation of Smad1 by increasing its dephosphorylation.[13]

Autophagy/Apoptosis module

The components and reactions for this module are shown in Figure S5. We assume that lysosome activity requires Beclin.[14] Beclin activity is inhibited when bound to Bcl2. In addition Beclin may be inactivated by an active caspase. The inactive form of Beclin can also bind to Bcl2. We assume that caspase is activated by pp38, Bax, or inactive Beclin, and that the caspase is inactivated by Bcl2. The model does not currently include details of apoptosis but levels of active caspase can be used as a marker to indicate a high probability of cell

death. Bcl2 degradation is increased by ROS[15] or active caspase which leads to increase pools of unbound Beclin. Bax activity is inhibited by Bcl2 which forms a complex with Bax. Bcl2 can also form complexes with Bax when it is bound to Beclin.

Figure S1 Damage module

Advanced glycation end-products (AGEprod) are spontaneously produced at a very low rate but accumulate with age. They activate RAGE receptors which increase levels of reactive oxygen species (ROS). ROS cause damage to proteins (DamP), lysosomes (Lys_I), and activates p38. DamP is removed by activated lysosomes (Lys_A). RAGE also activates NFκB (see Figure S2) which increases levels of SOD and so helps to reduce ROS. Aggrecan protects Collagen 2 from degradation (represented by complex Aggrecan_Collagen2). ADAMTS-5 cleaves Aggrecan to produce Aggrecan fragments (AggFrag) and releases Collagen 2. Collagen 2 is cleaved by MMP-13 to produce fragments (ColFrag).

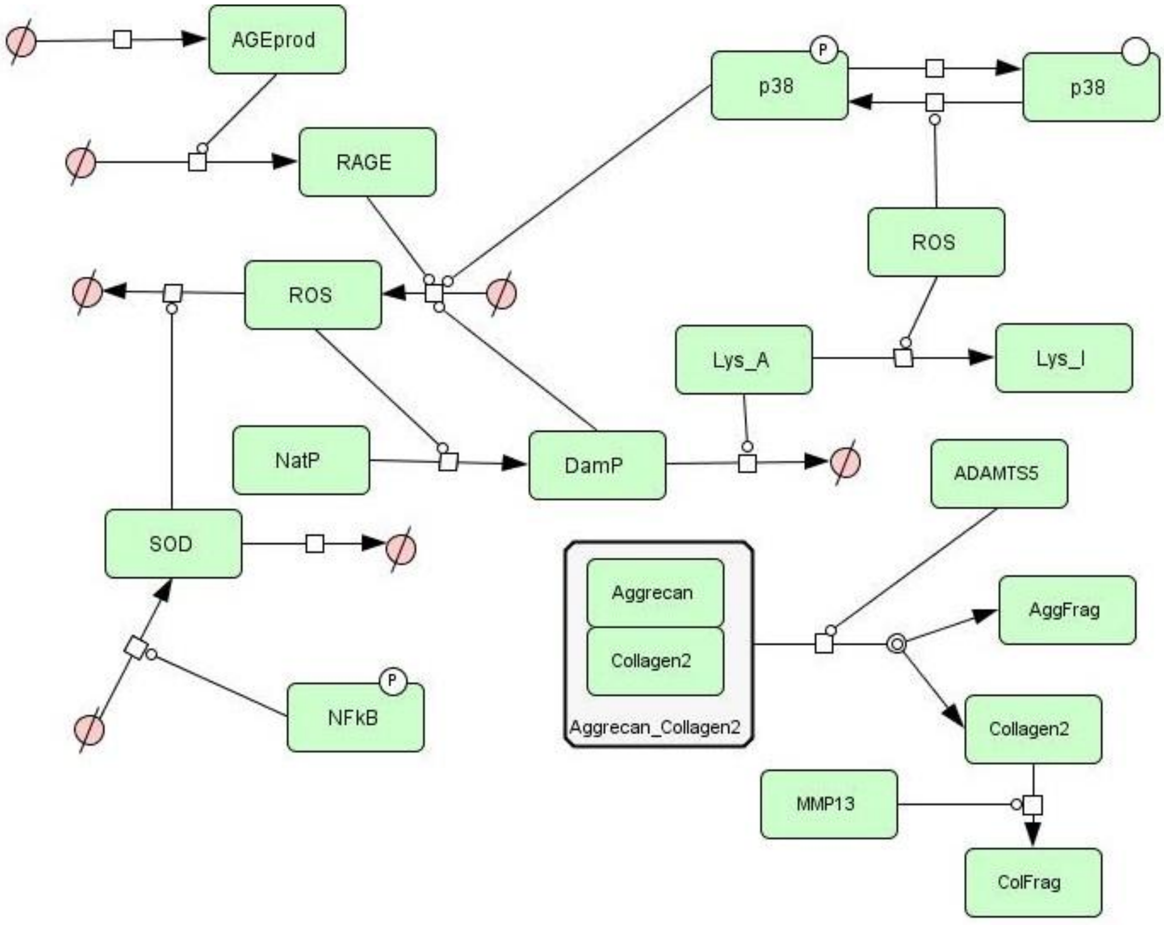

Key:

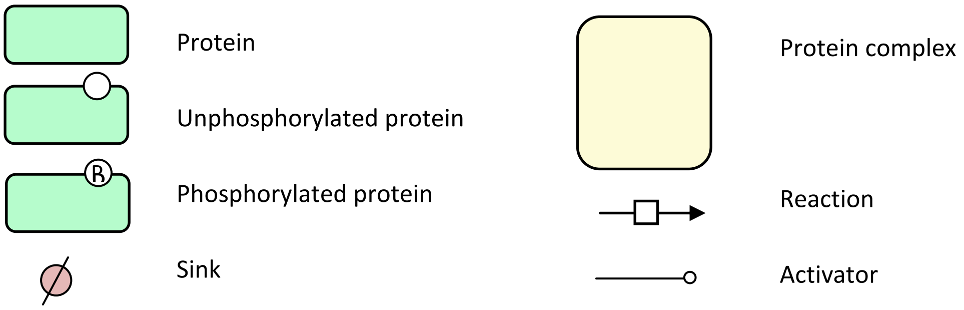

Figure S2 NFκB module

NFκB is normally inactive by being in complex with IκB (IkB_NFkB). Under stress conditions IκB is degraded releasing NFκB; in the model this can be catalysed by ROS or IL-1. IL-1 signalling leads to upregulation of ADAMTS-5, MMP-2 and MMP-13, and phosphorylation of p38. Pp38 phosphorylates NFκB which is then transcribes IL-1, IκB, RAGE and SOD (shown in Fig S1).

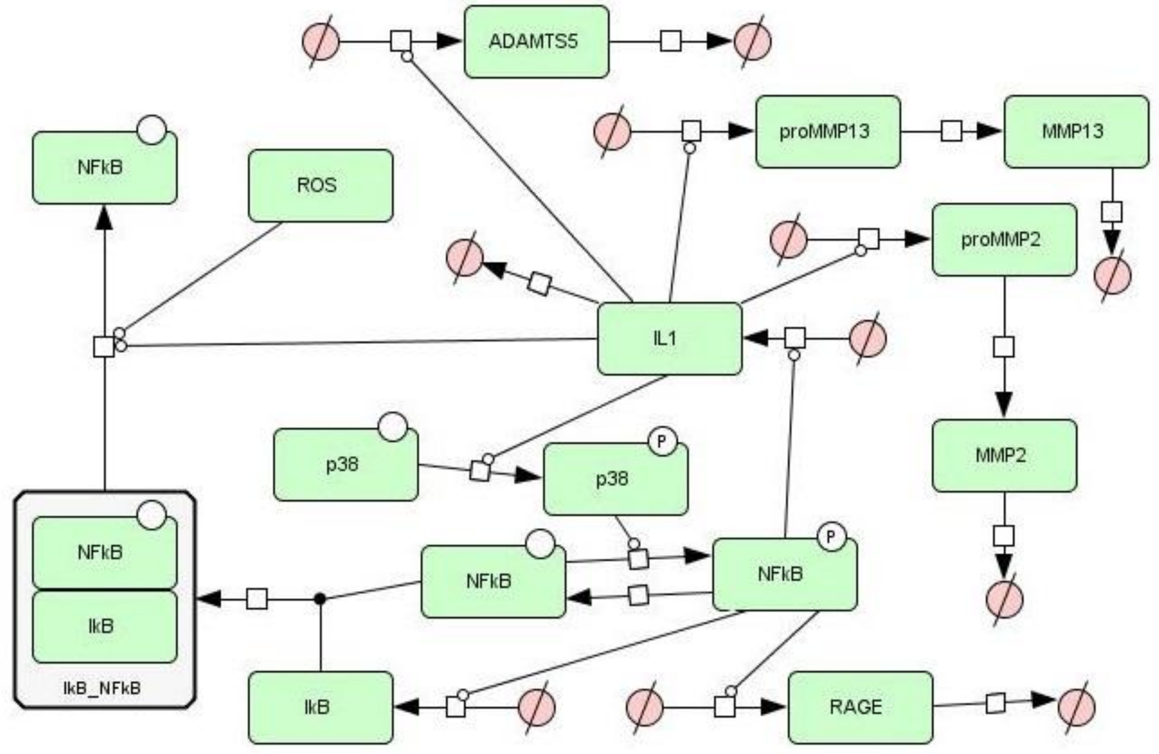

Key

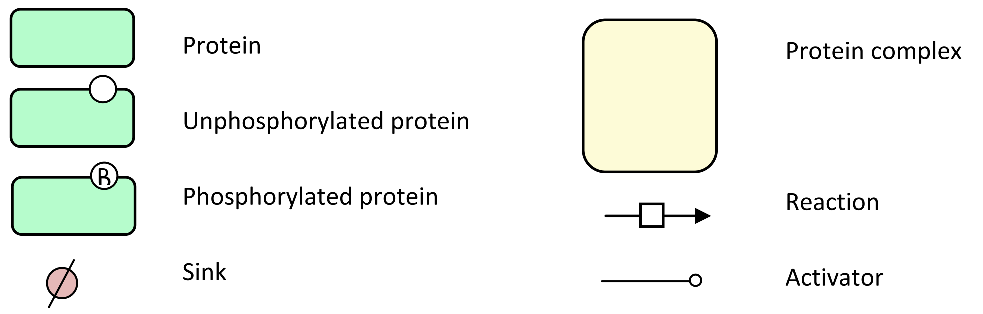

Figure S3 TGFβ/Alk5 module

Tgfβ is normally in the extra-cellular matrix in an inactive state (Tgfb_I). It can be activated by Integrin (which represents a mechanical stimuli) or MMP-2. To model a mechanical stimuli we assume that Integrin synthesis occurs at a very low rate and is then degraded very quickly. During the short time period when Integrin is present it can activate Tgfβ. Tgfβ signals via the Alk5 pathway by binding to Alk5 dimers. This leads to phosphorylation of Smad2 which then binds Smad4 (Smad2_P_Smad4). This complex activates Sox9 and also directly upregulates Collagen 2 and Smad7. Activated Sox9 (Sox9_A) upregulates Aggrecan, Collagen2 and Sox9. Smad7 binds to the Tgfβ/Alk5 complex which leads to degradation of Alk5 and Smad7 itself. We assume that Aggrecan binds to Collagen 2 to protect it from cleavage by MMPs.

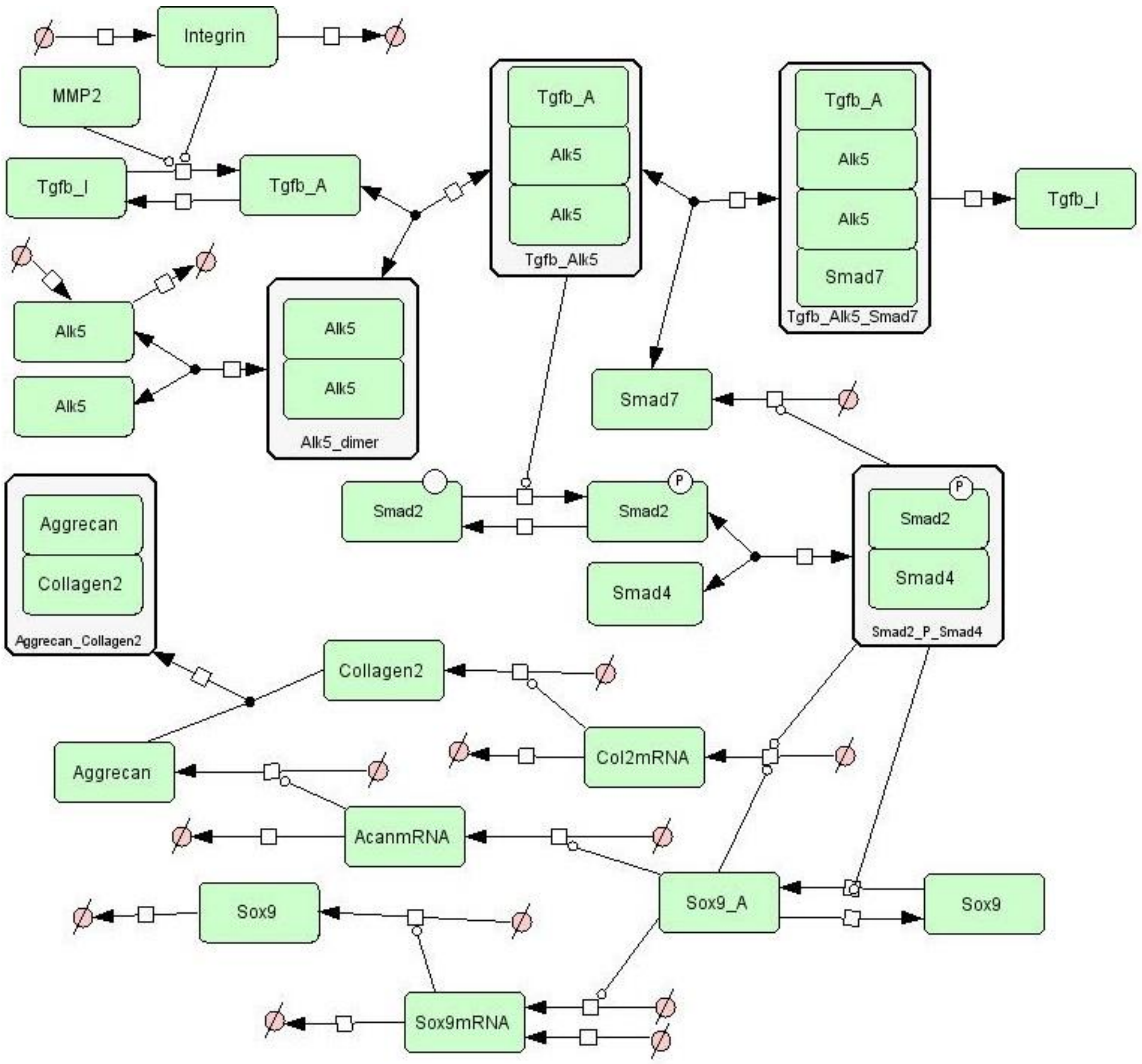

Key

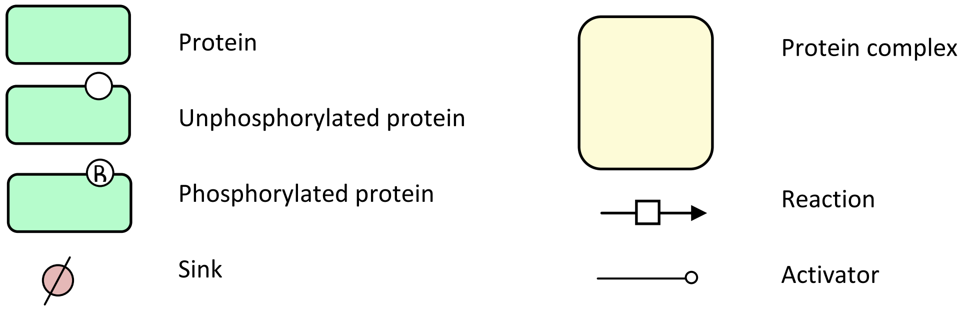

Figure S4 TGFβ/Alk1 module

Tgfβ also signals via the Alk1 pathway but this also requires Alk5. This is modelled by assuming that Alk1 forms a heterodimer with Alk5 before Tgfβ can bind. The Tgfβ/Alk1/Alk5 complex activates Smad1 by phosphorylation (Smad1_P). Smad1_P binds to Smad4 and upregulates Runx2 which then leads to upregulation of MMP-13. Smad2_P_Smad4 inhibits Runx2 activity. Smad7 may also bind to the Tgfβ/Alk1/Alk5 complex leading to degradation of Alk1, Alk5 and Smad7 itself. Smad7 also prevents activation of Smad1 by increasing its dephosphorylation.

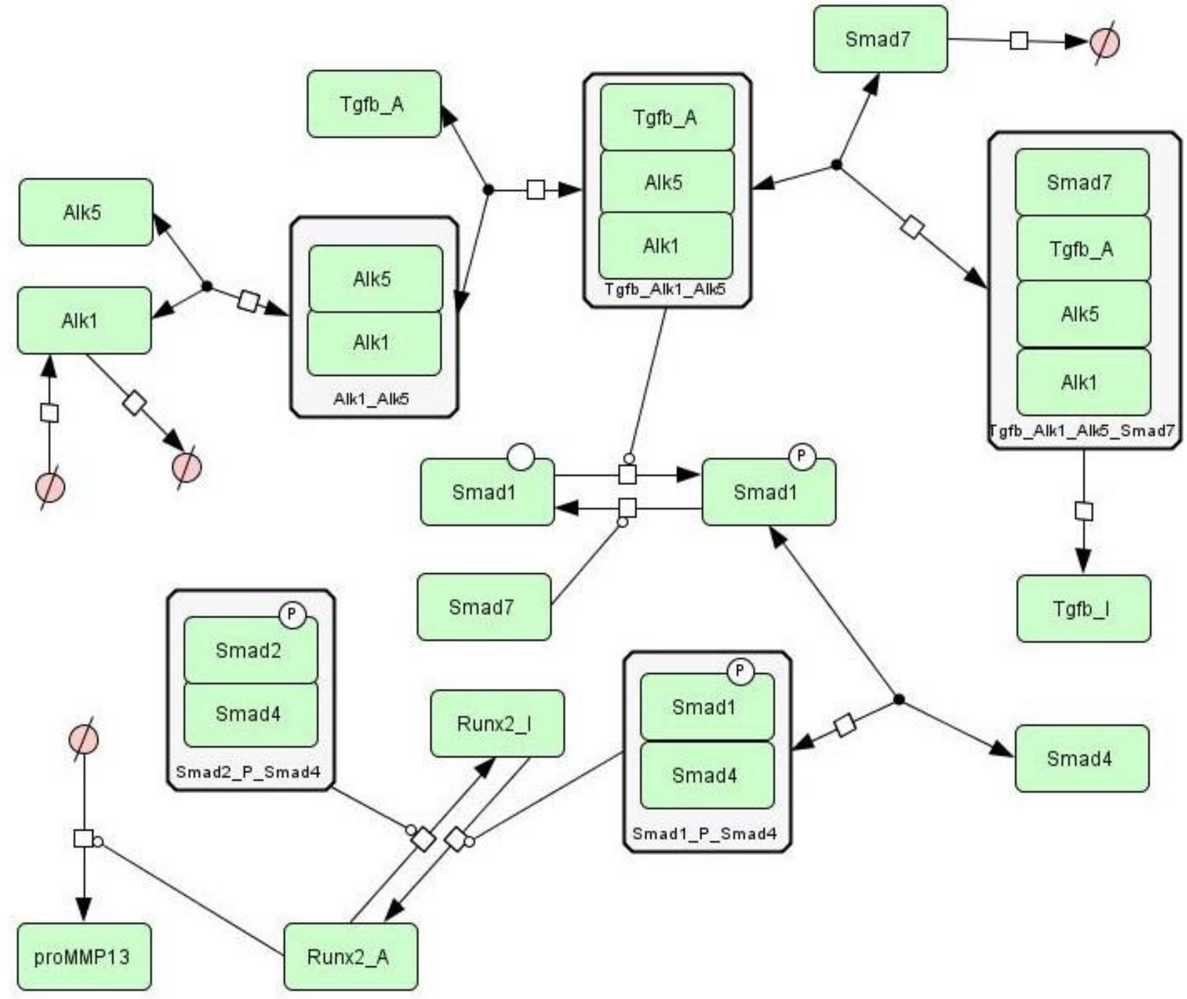

Key

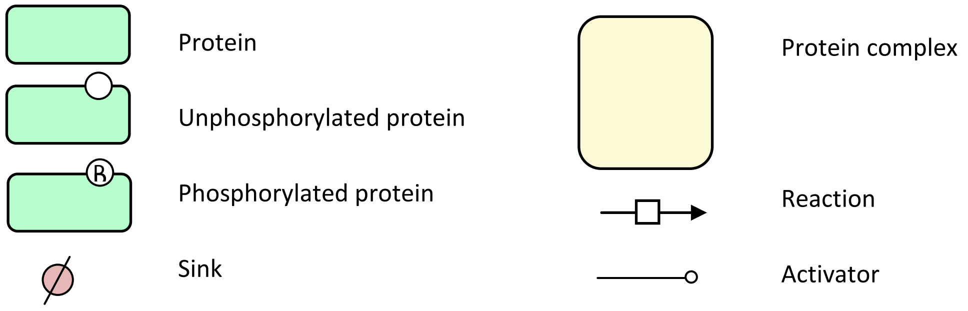

Figure S5 Autophagy/Apoptosis module

We assume that lysosome activity requires Beclin. Beclin activity is inhibited when bound to Bcl2. In addition Beclin may be inactivated by active Caspase (Caspase_A). The inactive form of Beclin is represented by Beclin_I and this can also bind to Bcl2. We assume that Caspase is activated by pp38, Bax, or Beclin_I and that it is inactivated by Bcl2. Bcl2 degradation is increased by ROS or Caspase_A which leads to increase pools of unbound Beclin. Bax activity is inhibited by Bcl2 which forms a complex with Bax. Bcl2 can also form complexes with Bax when it is bound to Beclin.

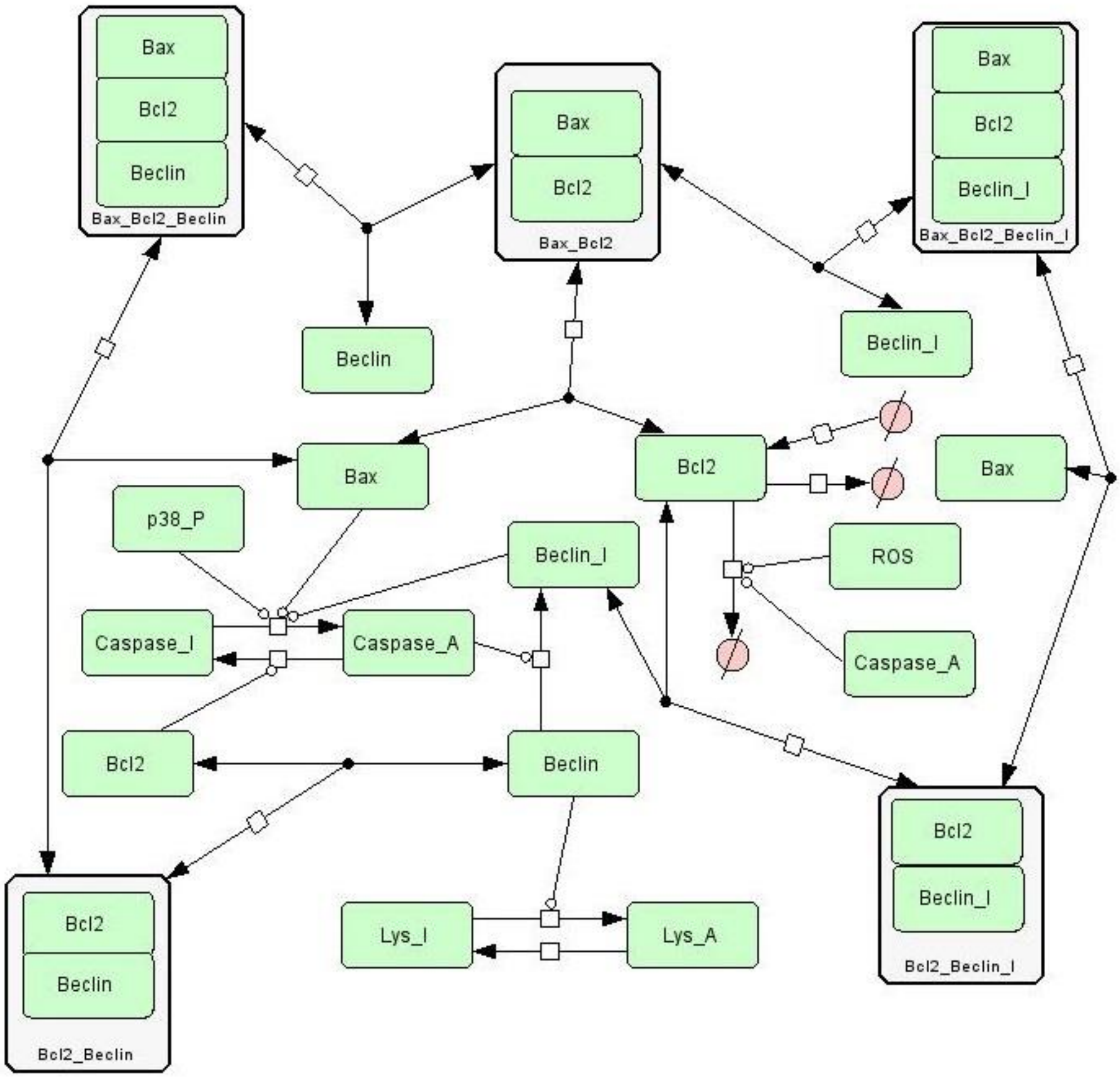

Key

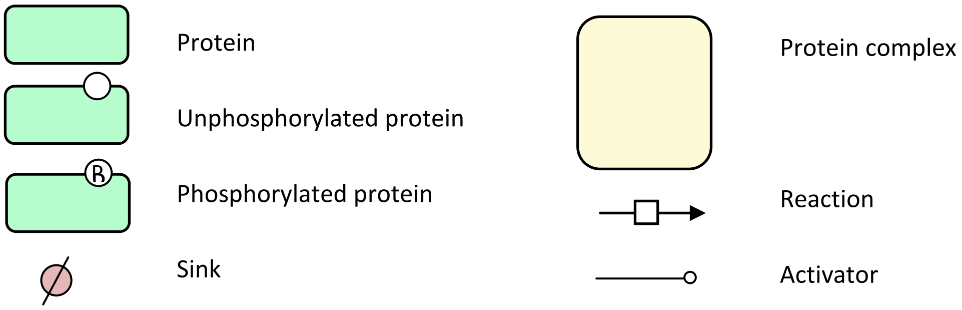

Table S1 List of model species

Name	Description	Database term	Initial amount	Relevant Figure(s)
AcanmRNA	Aggrecan messenger RNA	P16112	0	S3
ADAMTS5	A disintegrin and metalloproteinase with thrombospondin motifs 5	Q9UNA0	0	S1, S2
AGEprod	Advanced glycation end-products	-	0	S1
Aggrecan	Proteoglycan, component of the extracellular matrix (this is bound to collagen in model).	P16112	0	S3
AggFrag	Species to represent aggrecan fragments	-	0	S1
Aggrecan_collagen2	Complex to represent protection of collagen 2 by aggrecan	P16112, P02458	1000	S1, S3
Alk1	Serine/threonine-protein kinase receptor R3, Activin receptor-like kinase 1	P37023	500	S4
Alk1_Alk5	Alk1/Alk5 complex	P37023, P36897	0	S4
Alk5	TGF-beta receptor type 1, Activin receptor-like kinase 5	P36897	500	S3, S4
Alk5_dimer	Alk5 homodimer	P36897	0	S3
Bax	Apoptosis regulator	Q07812	0	S5
Bax_Bcl2	Complex of Bax and Bcl2	Q07812, P10415	90	S5
Bax_Bcl2_Beclin	Complex of Bax, Bcl2 and Beclin-1	Q07812, P10415, Q14457	10	S5
Bax_Bcl2_Beclin_I	Complex of Bax, Bcl2 and cleaved Beclin-1	Q07812, P10415, Q14457	0	S5
Bcl2	Apoptosis regulator (anti-apoptotic)	P10415	30	S5
Bcl2_Beclin	Complex of Bcl2 and Beclin-1	P10415, Q14457	25	S5
Bcl2_Beclin_I	Complex of Bcl2 and cleaved Beclin-1	P10415, Q14457	0	S5
Beclin	Beclin-1 protein, activator of autophagy	Q14457	75	S5
Beclin_I	Inactive Beclin (cleaved by Caspase)	Q14457	0	S5
Caspase_A	Caspase-3, activator of apoptosis	P42574	0	S5
Caspase_I	Inactive Caspase-3	P42574	100	S5
Col2mRNA	Collagen 2 messenger RNA	P02458	0	S3
Collagen2	Collagen 2, component of the extracellular matrix. This is unprotected pool.	P02458	0	S1, S3
ColFrag	Species to represent collagen fragment	-	0	S1
DamP	Damaged protein	-	0	S1
IkB	NFκB inhibitor	P25963	0	S2
IkB_NFkB	Complex of IκB and NFκB (inhibits NFκB)	P25963, Q04206	100	S2
IL1	Cytokine – interleukin-1α	P01583	0	S2
Integrin	Generic sensor of mechanical stress	-	0	S3
Lys_A	Active lysosome	GO:0005764	40	S1, S5
Lys_I	Inactive lysosome	GO:0005764	360	S1, S5
MMP13	Matrix metalloproteinase-13	P45452	0	S2
MMP2	Matrix metalloproteinase-2	P08253	0	S2, S3
NatP	Generic pool of native protein	-	1500	S1
NFkB	Transcription factor p65 (RelA)	Q04206	0	S2
NFkB_P	Phosphorylated p65	Q04206	0	S1, S2
P38	P38 MAPK kinase (MAPK14)	Q16539	100	S1, S2
P38_P	Phosphorylated p38	Q16539	0	S1, S2, S5
proMMP13	Inactive form of MMP-13	P45452	0	S2, S4
ProMMP2	Inactive form of MMP-2	P08253	0	S2
RAGE	Receptor for AGE products	Q15109	0	S1, S2
ROS	Reactive oxygen species	CHEBI:26523	2	S1, S2, S5

Runx2_I	Inactive runt-related transcription factor 2	Q13950	100	S4
Runx2_A	Active Runx2	Q13950	0	S4
Smad2	Mothers against decapentaplegic homolog2	Q15796	600	S3
Smad2_P	Phosphorylated Smad2	Q15796	0	S3
Smad4	Mothers against decapentaplegic homolog4	Q13485	600	S3, S4
Smad2_P_Smad4	Complex of Smad2 and Smad4	Q15796, Q13485	0	S3, S4
Smad1	Mothers against decapentaplegic homolog1	Q15797	600	S4
Smad1_P	Phosphorylated Smad1	Q15797	0	S4
Smad1_P_Smad4	Complex of Smad1 and Smad4	Q15797, Q13485	0	S4
Smad7	Mothers against decapentaplegic homolog7	O15105	0	S3, S4
SOD	Superoxide dismutase 1	P00441	2	S1
Sox9	Transcription factor Sox-9	P48436	100	S3
Sox9_A	Activated Sox9	P48436	0	S3
Sox9mRNA	Sox9 messenger RNA	P48436	10	S3
Tgfb_A	Active transforming growth factor beta-1	P01137	0	S3, S4
Tgfb_Alk1_Alk5	Tgfβ bound to Alk1/Alk5 heterodimer	P01137, P37023, P36897	0	S4
Tgfb_Alk5_dimer	Tgfβ bound to Alk5 homodimer	P01137, P36897	0	S3
Tgfb_Alk1_Alk5_Smad7	Smad7 bound to Tgfβ/Alk1/Alk5 complex	P01137, P37023, P36897, O15105	0	S4
Tgfb_Alk5_dimer_Smad7	Smad7 bound to Tgfβ/Alk5 complex	P01137, P36897, O15105	0	S3
Tgfb_I	Inactive Tgfβ	P01137	200	S3, S4

Table S2 Reactions for Damage module (all reactions are shown in Figure S1)

Reaction id	Reactants and products	Kinetic rate law	Parameter value[a]
ROS_generation	Source → ROS	k_{genROS} * Source	5.0e-4 mol s^{-1}
ROS_removal	ROS → Sink	k_{remROS} * ROS	3.83e-4 s^{-1}
Protein_damage_by_ROS	NatP+ROS → DamP+ROS	$k_{damNatP}$ * NatP * ROS / (10 + ROS)	8.0e-6 s^{-1}
Removal_DamP_by_Autophagy	DamP+Lys_A → Lys_A	$k_{degDamP}$ * DamP * Lys_A	4.0e-5 $mol^{-1}s^{-1}$
Production_of_AGE_Products	Source → AGEprod	$k_{prodAGE}$ * Source	1.0e-6 mol s^{-1}
RAGE_activation	AGEprod → AGEprod+RAGE	$k_{actRAGE}$ * AGEprod	1.0e-3 s^{-1}
ROS_production_by_RAGE	RAGE → RAGE+ROS	$k_{genROSbyRAGE}$ * RAGE	4.0e-4 s^{-1}
Aggrecan_degradation	Aggrecan_Collagen2+ADAMTS5 → Collagen2+ADAMTS5+AggFrag	$k_{degAggrecan}$ * Aggrecan_Collagen2 * ADAMTS5	1.0e-9 $mol^{-1}s^{-1}$
Collagen_degradation	Collagen2+MMP13 → MMP13+ColFrag	$k_{degCollagen}$ * Collagen2 * MMP13	1.0e-8 $mol^{-1}s^{-1}$
ROS_production_by_DamP	DamP → DamP+ROS	$k_{genROSbyDamP}$ * DamP	1.0e-4 s^{-1}
SOD_synthesis	NFkB_P → NFkB_P+SOD	k_{synSOD} * NFkB_P	2.0e-3 s^{-1}
SOD_degradation	SOD → Sink	k_{degSOD} * SOD	1.0e-3 s^{-1}
ROS_removal_by_SOD	ROS+SOD → SOD	$k_{remROSbySOD}$ * SOD * ROS	1.0e-4 $mol^{-1}s^{-1}$
p38_phosphorylation	p38+IL1 → p38_P+IL1	$k_{phosp38}$ * p38 * IL1	1.0e-7 $mol^{-1}s^{-1}$
p38_phosphorylation_via_ROS	p38+ROS → p38_P+ROS	$k_{phosp38ROS}$ * p38 * ROS	1.0e-4 $mol^{-1}s^{-1}$
p38_dephosphorylation	p38_P → p38	$k_{dephosp38}$ * p38_P	0.01 s^{-1}
ROS_production_by_p38_P	p38_P → p38_P+ROS	$k_{genROSbyp38}$ * p38_P	1.0e-4 s^{-1}
Lysosome_damage_by_ROS	Lys_A+ROS → Lys_I+ROS	k_{damLys} * Lys_A * ROS / (10 + ROS)	5.0e-6 s^{-1}

[a] mol=number of molecules

Table S3 Reactions for NFκB module (all reactions are shown in Figure S2)

Reaction id	Reactants and products	Kinetic rate law	Parameter value[a]
IkB_degradation_via_ROS	ROS+IkB_NFkB → ROS+NFkB	k_{degIkB} * ROS * IkB_NFkB	1.0e-6 $mol^{-1}s^{-1}$
IkB_degradation_via_IL1	IL1+IkB_NFkB → IL1+NFkB	k_{degIkB} * IL1 * IkB_NFkB	1.0e-6 $mol^{-1}s^{-1}$
NFkB_inactivation	NFkB+IkB → IkB_NFkB	$k_{inactNFkB}$ * NFkB * IkB	0.1 $mol^{-1}s^{-1}$
RAGE_inactivation	RAGE → Sink	$k_{inactRAGE}$ * RAGE	1.0e-3 s^{-1}
RAGE_upregulation_by_ NFkB	NFkB_P → NFkB_P+RAGE	$k_{synRAGE}$ * NFkB_P	1.0e-4 s^{-1}
IL1_production	NFkB_P → NFkB_P+IL1	k_{synIL1} * NFkB_P	5.0e-3 s^{-1}
IL1_degradation	IL1 → Sink	k_{degIL1} * IL1	5.0e-3 s^{-1}
IkB_production	NFkB_P → NFkB_P+IkB	k_{synIkB} * NFkB_P	1.0e-3 s^{-1}
MMP13_production	IL1 → IL1+proMMP13	$k_{synMMP13}$ * IL1	3.2e-5 s^{-1}
MMP13_activation	proMMP13 → MMP13	$k_{actMMP13}$ * proMMP13	1.0e-4 s^{-1}
MMP13_removal	MMP13 → Sink	$k_{degMMP13}$ * MMP13	6.4e-6 s^{-1}
MMP2_production	IL1 → IL1+proMMP2	$k_{synMMP2}$ * IL1	5.0e-6 s^{-1}
MMP2_activation	proMMP2 → MMP2	$k_{actMMP2}$ * proMMP2	1.0e-7 s^{-1}
MMP2_degradation	MMP2 → Sink	$k_{degMMP2}$ * MMP2	6.4e-6 s^{-1}
ADAMTS5_production	IL1 → IL1+ADAMTS5	$k_{synADAMTS5}$ * IL1	5.0e-4 s^{-1}
ADAMTS5_removal	ADAMTS5 → Sink	$k_{degADAMTS5}$ * ADAMTS5	5.0e-5 s^{-1}
NFkB_activation	NFkB+p38_P → NFkB_P+p38_P	$k_{phosNFkB}$ * NFkB * p38_P	1.0e-3 $mol^{-1}s^{-1}$
NFkB_dephosphorylation	NFkB_P → NFkB	$k_{dephosNFkB}$ * NFkB_P	0.01 s^{-1}

[a] mol=number of molecules

Table S4 Reactions for TGFβ/Alk5 module (all reactions are shown in Figure S3)

Reaction id	Reactants and products	Kinetic rate law	Parameter value[a]
Integrin_activation	Source → Integrin +IntegrinCount	$k_{actIntegrin}$ * Source	4.0e-7 mol s^{-1}
Integrin_inactivation	Integrin → Sink	$k_{inactIntegrin}$ * Integrin	5.0e-4 s^{-1}
Alk5_synthesis	Source → Alk5	$k_{synAlk5}$ * Source	5.0e-6 mol s^{-1}
Tgfb_activation_by_ integrin	Tgfb_I+Integrin → Tgfb_A+Integrin	$k_{actTgfbIntegrin}$ * Tgfb_I * Integrin	1.0e-3 $mol^{-1}s^{-1}$
Tgfb_activation_by_MMP2	Tgfb_I+MMP2 → Tgfb_A+MMP2	$k_{actTgfbMMP2}$ * Tgfb_I * MMP2	1.0e-7 $mol^{-1}s^{-1}$
Tgfb_inactivation	Tgfb_A → Tgfb_I	$k_{inactTgfb}$ * Tgfb_A	0.05 s^{-1}
Alk5_dimerisation	2Alk5 → Alk5_dimer	$k_{dimerAlk5}$ * Alk5 * (Alk5 - 1) * 0.5	2.0e-4 $mol^{-1}s^{-1}$
Alk5_dedimerisation	Alk5_dimer → 2Alk5	$k_{dedimerAlk5}$ * Alk5_dimer	1.0e-3 s^{-1}
Tgfb_Alk5_binding	Tgfb_A+Alk5_dimer → Tgfb_Alk5_dimer	$k_{binTgfbAlk5}$ * Tgfb_A * Alk5_dimer	3.0e-5 $mol^{-1}s^{-1}$
Tgfb_Alk5_release	Tgfb_Alk5_dimer → Tgfb_A+Alk5_dimer	$k_{relTgfbAlk5}$ * Tgfb_Alk5_dimer	1.0e-6 s^{-1}
Tgfb_Alk5_Smad7_binding	Tgfb_Alk5_dimer+Smad7 → Tgfb_Alk5_dimer_Smad7	$k_{binSmad7Alk5}$ * Tgfb_Alk5_dimer * Smad7	2.0e-5 $mol^{-1}s^{-1}$
Tgfb_Alk5_Smad7_release	Tgfb_Alk5_dimer_Smad7 → Tgfb_Alk5_dimer+Smad7	$k_{relSmad7Alk5}$ * Tgfb_Alk5_dimer_Smad7	1.0e-6 s^{-1}
Alk5_Smad7_degradation	Tgfb_Alk5_dimer_Smad7 → Tgfb_I	$k_{degSmad7Alk5}$ * Tgfb_Alk5_dimer_Smad7	1.0e-5 s^{-1}
Smad2_phosphorylation	Tgfb_Alk5_dimer+Smad2 → Tgfb_Alk5_dimer +Smad2_P	$k_{phosSmad2}$ * Tgfb_Alk5_dimer * Smad2	4.0e-5 $mol^{-1}s^{-1}$
Smad2_Smad4_binding	Smad2_P+Smad4 → Smad2_P_Smad4	$k_{binSmad2Smad4}$ * Smad2_P * Smad4	1.0e-4 $mol^{-1}s^{-1}$
Smad2P_Smad4_release	Smad2_P_Smad4 → Smad2_P+Smad4	$k_{relSmad2Smad4}$ * Smad2_P_Smad4	0.0167 s^{-1}
Smad2_dephosphorylation	Smad2_P → Smad2	$k_{dephosSmad2}$ * Smad2_P	6.0e-3 s^{-1}
Smad7_synthesis	Smad2_P_Smad4 → Smad2_P_Smad4+Smad7	$k_{synSmad7}$ * Smad2_P_Smad4	1.0e-5 s^{-1}
Sox9_activation	Smad2_P_Smad4 +Sox9 → Smad2_P_Smad4 +Sox9_A	$k_{actSox9}$ * Smad2_P_Smad4 * Sox9	5.0e-6 $mol^{-1}s^{-1}$
Sox9_inactivation	Sox9_A → Sox9	$k_{inactSox9}$ * Sox9_A	1.5e-3 s^{-1}
Sox9_basal_transcription	Source → Sox9mRNA	$k_{synSox9mRNA}$ * Source	1.0e-5 mol s^{-1}
Sox9_enhanced_ transcription	Sox9_A → Sox9_A+Sox9mRNA	$k_{synSox9mRNASox9A}$ * Sox9_A	5.0e-6 s^{-1}

Sox9mRNA_degradation	Sox9mRNA → Sink	$k_{degSox9mRNA}$ * Sox9mRNA	1.0e-4 s^{-1}
Sox9_translation	Sox9mRNA → Sox9mRNA+Sox9	$k_{synSox9}$ * Sox9mRNA	4.8e-4 s^{-1}
Sox9_degradation	Sox9 → Sink	$k_{degSox9}$ * Sox9	4.8e-5 s^{-1}
Collagen2_enhanced_ transcription_by_Sox9	Sox9_A → Sox9_A+Col2mRNA	$k_{synCol2mRNASox9A}$ * Sox9_A	1.0e-6 s^{-1}
Collagen2_transcription_by Smad2_Smad4	Smad2_P_Smad4 → Smad2_P_Smad4 +Col2mRNA	$k_{synCol2mRNASmad}$ * Smad2_P_Smad4	1.0e-6 s^{-1}
Col2mRNA_degradation	Col2mRNA → Sink	$k_{degCol2mRNA}$ * Col2mRNA	1.0e-7 s^{-1}
Collagen2_translation	Col2mRNA → Col2mRNA+Collagen2	$k_{synCol2}$ * Col2mRNA	1.0e-7 s^{-1}
Aggrecan_transcription	Sox9_A → Sox9_A+AcanmRNA	$k_{synAcanmRNASox9A}$ * Sox9_A	4.6e-6 s^{-1}
AcanmRNA_degradation	AcanmRNA → Sink	$k_{degAcanmRNA}$ * AcanmRNA	9.0e-6 s^{-1}
Aggrecan_translation	AcanmRNA → AcanmRNA+Aggrecan	$k_{synAggrecan}$ * AcanmRNA	1.0e-6 s^{-1}
Aggrecan_Collagen2_ binding	Aggrecan+Collagen2 → Aggrecan_Collagen2	$k_{binAggrecanCollagen2}$ * Aggrecan * Collagen2	1.0e-4 $mol^{-1}s^{-1}$
Alk5_degradation	Alk5 → Sink	$k_{degAlk5}$ * Alk5	4.0e-7 s^{-1}

[a] mol=number of molecules

Table S5 Reactions for TGFβ/Alk1 module (all reactions are shown in Figure S4)

Reaction id	Reactants and products	Kinetic rate law	Parameter value[a]
Alk1_Alk5_binding	Alk1+Alk5 → Alk1_Alk5	$k_{binAlk1Alk5}$ * Alk1 * Alk5	5.0e-5 $mol^{-1}s^{-1}$
Alk1_Alk5_release	Alk1_Alk5 → Alk1+Alk5	$k_{relAlk1Alk5}$ * Alk1_Alk5	0.01 s^{-1}
Tgfb_Alk1_binding	Tgfb_A+Alk1_Alk5 → Tgfb_Alk1_Alk5	$k_{binTgfbAlk1}$ * Tgfb_A * Alk1_Alk5	2.0e-5 $mol^{-1}s^{-1}$
Tgfb_Alk1_release	Tgfb_Alk1_Alk5 → Tgfb_A+Alk1_Alk5	$k_{relTgfbAlk1}$ * Tgfb_Alk1_Alk5	1.0e-6 s^{-1}
Runx2_inhibition_by_Smad2	Runx2_A +Smad2_P_Smad4 → Runx2_I+Smad2_P_Smad4	$k_{inactRunx2}$ * Runx2_A * Smad2_P_Smad4	5.0e-4 $mol^{-1}s^{-1}$
Smad1_activation	Tgfb_Alk1_Alk5+Smad1 → Tgfb_Alk1_Alk5+Smad1_P	$k_{phosSmad1}$ * Tgfb_Alk1_Alk5 * Smad1	2.0e-5 $mol^{-1}s^{-1}$
Smad1_dephosphorylation	Smad1_P → Smad1	$k_{dephosSmad1}$ * Smad1_P	5.0e-4 s^{-1}
Smad1_dephosphorylation_ via_Smad7	Smad1_P+Smad7 → Smad1+Smad7	$k_{dephosSmad1Smad7}$ * Smad1_P * Smad7	6.0e-4 $mol^{-1}s^{-1}$
Smad1_Smad4_binding	Smad1_P+Smad4 → Smad1_P_Smad4	$k_{binSmad1Smad4}$ * Smad1_P * Smad4	5.0e-5 $mol^{-1}s^{-1}$
Smad1_Smad4_release	Smad1_P_Smad4 → Smad1_P+Smad4	$k_{relSmad1Smad4}$ * Smad1_P_Smad4	0.0167 s^{-1}
Runx2_activation_by_Smad1	Runx2_I+ Smad1_P_Smad4 → Runx2_A +Smad1_P_Smad4	$k_{actRunx2}$ * Runx2_I * Smad1_P_Smad4	1.0e-3 $mol^{-1}s^{-1}$
MMP13_induction_by_ Runx2	Runx2_A → proMMP13+Runx2_A	$k_{synMMP13Runx2}$ * Runx2_A	1.5e-6 s^{-1}
Alk1_synthesis	Source → Alk1	$k_{synAlk1}$ * Source	5.0e-6 mol s^{-1}
Alk1_degradation	Alk1 → Sink	$k_{degAlk1}$ * Alk1	1.2e-8 s^{-1}
Tgfb_Alk1_Alk5_Smad7_ binding	Tgfb_Alk1_Alk5+Smad7 → Tgfb_Alk1_Alk5_Smad7	$k_{binSmad7Alk1}$ * Tgfb_Alk1_Alk5 * Smad7	0.5 $mol^{-1}s^{-1}$
Tgfb_Alk1_Alk5_ Smad7_release	Tgfb_Alk1_Alk5_Smad7 → Tgfb_Alk1_Alk5+Smad7	$k_{relSmad7Alk1}$ * Tgfb_Alk1_Alk5_Smad7	1.0e-3 s^{-1}
Alk1_Smad7_degradation	Tgfb_Alk1_Alk5_Smad7 → Tgfb_I	$k_{degSmad7Alk1}$ * Tgfb_Alk1_Alk5_Smad7	5.0e-6 s^{-1}
Smad7_degradation	Smad7 → Sink	$k_{degSmad7}$ * Smad7	5.0e-3 s^{-1}

[a] mol=number of molecules

Table S6 Reactions for Autophagy/Apoptosis module (all reactions are shown in Figure S5)

Reaction id	Reactants and products	Kinetic rate law	Parameter value[a]
Caspase_activation	Caspase_I+Bax → Caspase_A+Bax	$k_{actCasp}$ * Caspase_I * Bax	1e-7 $mol^{-1}s^{-1}$
Caspase_activation_by_Beclin_I	Caspase_I+Beclin_I → Caspase_A+Beclin_I	$k_{actCaspBec\ I}$* Caspase_I * Beclin_I	8.3e-7 $mol^{-1}s^{-1}$
Caspase_activation_by_p38	Caspase_I+p38_P → Caspase_A+p38_P	$k_{actCaspp38}$ * Caspase_ I* p38_P	8.0e-7 $mol^{-1}s^{-1}$
Caspase_inactivation	Caspase_A → Caspase_I	$k_{inactCasp}$ * Caspase_A	3.0e-4 s^{-1}
Caspase_inactivation_by_Bcl2_Beclin	Caspase_A+Bcl2_Beclin → Caspase_I+Bcl2_Beclin	$k_{inactCaspBcl2}$ * Caspase_A * Bcl2_Beclin	3.0e-4 $mol^{-1}s^{-1}$
Caspase_inactivation_by_Bcl2	Caspase_A+Bcl2 → Caspase_I+Bcl2	$k_{inactCaspBcl2}$ * Caspase_A * Bcl2	3.0e-4 $mol^{-1}s^{-1}$
Lysosome_activation	Lys_I+Beclin → Lys_A+Beclin	k_{actLys}*Lys_I*Beclin	1.0e-8 $mol^{-1}s^{-1}$
Lysosome_inhibition	Lys_A → Lys_I	$k_{inhibLys}$*Lys_A	7.0e-6 s^{-1}
Bcl2_synthesis	Source → Bcl2	$k_{synBcl2}$ * Source	2.0e-3 mol s^{-1}
Bcl2_degradation	Bcl2 → Sink	$k_{degBcl2}$ * Bcl2	1.67e-4 s^{-1}
Bcl2_degradation_induced_by_stress	Bcl2+ROS → Sink+ROS	$k_{degBcl2ROS}$ * Bcl2 * ROS	1.67e-3 $mol^{-1}s^{-1}$
Bcl2_degradation_induced_by_caspase	Bcl2+Caspase_A → Sink+Caspase_A	$k_{degBcl2Casp}$ * Bcl2 * Caspase_A	1.67e-3 $mol^{-1}s^{-1}$
Bax_Bcl2_binding	Bax+Bcl2 → Bax_Bcl2	$k_{binBaxBcl2}$ * Bax * Bcl2	1.67 $mol^{-1}s^{-1}$
Bax_Bcl2_release	Bax_Bcl2 → Bax+Bcl2	$k_{relBaxBcl2}$ * Bax_Bcl2	1.67e-3 s^{-1}
Bcl2_Beclin_binding	Bcl2+Beclin → Bcl2_Beclin	$k_{binBcl2Beclin}$ * Bcl2 * Beclin	7.5e-5 $mol^{-1}s^{-1}$
Bcl2_Beclin_release	Bcl2_Beclin → Bcl2+Beclin	$k_{relBcl2Beclin}$ * Bcl2_Beclin	5.0e-4 s^{-1}
Bcl2_Beclin_I_binding	Bcl2+Beclin_I → Bcl2_Beclin_I	$k_{binBcl2Beclin}$ * Bcl2 * Beclin_I	7.5e-5 $mol^{-1}s^{-1}$
Bcl2_Beclin_I_release	Bcl2_Beclin_I → Bcl2+Beclin_I	$k_{relBcl2Beclin}$ * Bcl2_Beclin_I	5.0e-4 s^{-1}
Beclin_inactivation	Beclin → Beclin_I	$k_{inactBec}$ * Beclin	5.0e-10 s^{-1}
Beclin_inactivation_by_caspase	Beclin+Caspase_A → Beclin_I+Caspase_A	$k_{inactBecCasp}$ * Beclin * Caspase_A	1.0e-8 $mol^{-1}s^{-1}$
Beclin_ Bax_Bcl2_binding	Beclin+Bax_Bcl2 → Bax_Bcl2_Beclin	$k_{binBecToBaxBcl2}$ * Beclin * Bax_Bcl2	1.67e-5 $mol^{-1}s^{-1}$
Beclin_I_Bax_Bcl2_binding	Beclin_I+Bax_Bcl2 → Bax_Bcl2_Beclin_I	$k_{binBecToBaxBcl2}$ * Beclin_I* Bax_Bcl2	1.67e-5 $mol^{-1}s^{-1}$
Bax_Bcl2_Beclin_binding	Bax+Bcl2_Beclin → Bax_Bcl2_Beclin	$k_{binBaxToBcl2Bec}$ * Bax * Bcl2_Beclin	1.67e-4 $mol^{-1}s^{-1}$
Bax_Bcl2_Beclin_I_binding	Bax+Bcl2_Beclin_I → Bax_Bcl2_Beclin_I	$k_{binBaxToBcl2Bec}$ * Bax * cl2_Beclin_I	1.67e-4 $mol^{-1}s^{-1}$
Bax_dissociation_from_Bax_Bcl2_Beclin	Bax_Bcl2_Beclin → Bax+Bcl2_Beclin	$k_{relBaxBcl2Bec}$ * Bax_Bcl2_Beclin	1.67e-3 s^{-1}
Bax_dissociation_from_Bax_Bcl2_Beclin_I	Bax_Bcl2_Beclin_I → Bax+Bcl2_Beclin_I	$k_{relBaxBcl2Bec}$ * Bax_Bcl2_Beclin_I	1.67e-3 s^{-1}
Beclin_dissociation_from_Bax_Bcl2_Beclin	Bax_Bcl2_Beclin → Beclin+Bax_Bcl2	$k_{relBecBaxBcl2}$ * Bax_Bcl2_Beclin	1.67e-2 s^{-1}
Beclin_I_dissociation_from_Bax_Bcl2_Beclin	Bax_Bcl2_Beclin_I → Beclin_I+Bax_Bcl2	$k_{relBecBaxBcl2}$ * Bax_Bcl2_Beclin_I	1.67e-2 s^{-1}

[a] mol=number of molecules

References

1. Loeser RF, Yammani RR, Carlson CS, Chen H, Cole A, Im HJ, et al. Articular chondrocytes express the receptor for advanced glycation end products: Potential role in osteoarthritis. *Arthritis Rheum* 2005;52(8):2376-85.
2. Passos JF, Nelson G, Wang C, Richter T, Simillion C, Proctor CJ, et al. Feedback between p21 and reactive oxygen production is necessary for cell senescence. *Mol. Syst. Biol.* 2010;6:347.
3. Pratta MA, Yao W, Decicco C, Tortorella MD, Liu RQ, Copeland RA, et al. Aggrecan protects cartilage collagen from proteolytic cleavage. *The Journal of biological chemistry* 2003;278:45539 - 45.
4. Stanton H, Rogerson FM, East CJ, Golub SB, Lawlor KE, Meeker CT, et al. ADAMTS5 is the major aggrecanase in mouse cartilage in vivo and in vitro. *Nature* 2005;434:648 - 52.

5. Wang M, Sampson E, Jin H, Li J, Ke Q, Im H-J, et al. MMP13 is a critical target gene during the progression of osteoarthritis. *Arthrit. Res. Ther.* 2013;15(1):R5.
6. Largo R, Alvarez-Soria MA, Diez-Ortego I, Calvo E, Sanchez-Pernaute O, Egido J, et al. Glucosamine inhibits IL-1beta-induced NFkappaB activation in human osteoarthritic chondrocytes. *Osteoarthritis Cartilage* 2003;11(4):290-8.
7. Fan Z, Soder S, Oehler S, Fundel K, Aigner T. Activation of interleukin-1 signaling cascades in normal and osteoarthritic articular cartilage. *Am J Pathol* 2007;171(3):938-46.
8. Ulivi V, Giannoni P, Gentili C, Cancedda R, Descalzi F. p38/NF-kB-dependent expression of COX-2 during differentiation and inflammatory response of chondrocytes. *J. Cell. Biochem.* 2008;104(4):1393-406.
9. Sato Y, Rifkin DB. Inhibition of endothelial cell movement by pericytes and smooth muscle cells: activation of a latent transforming growth factor-beta 1-like molecule by plasmin during co-culture. *The Journal of cell biology* 1989;109(1):309-15.
10. Kavsak P, Rasmussen RK, Causing CG, Bonni S, Zhu H, Thomsen GH, et al. Smad7 Binds to Smurf2 to Form an E3 Ubiquitin Ligase that Targets the TGFβ Receptor for Degradation. *Mol. Cell* 2000;6(6):1365-75.
11. Finnson KW, Parker WL, ten Dijke P, Thorikay M, Philip A. ALK1 Opposes ALK5/Smad3 Signaling and Expression of Extracellular Matrix Components in Human Chondrocytes. *J. Bone Miner. Res.* 2008;23(6):896-906.
12. Chen CG, Thuillier D, Chin EN, Alliston T. Chondrocyte-intrinsic Smad3 represses Runx2-inducible matrix metalloproteinase 13 expression to maintain articular cartilage and prevent osteoarthritis. *Arthritis Rheum* 2012;64(10):3278-89.
13. Valdimarsdottir G, Goumans MJ, Itoh F, Itoh S, Heldin CH, ten Dijke P. Smad7 and protein phosphatase 1alpha are critical determinants in the duration of TGF-beta/ALK1 signaling in endothelial cells. *BMC Cell Biol* 2006;7:16.
14. Kang R, Zeh HJ, Lotze MT, Tang D. The Beclin 1 network regulates autophagy and apoptosis. *Cell Death Differ* 2011;18(4):571-80.
15. Azad N, Iyer AK, Manosroi A, Wang L, Rojanasakul Y. Superoxide-mediated proteasomal degradation of Bcl-2 determines cell susceptibility to Cr(VI)-induced apoptosis. *Carcinogenesis* 2008;29(8):1538-45.
